# Cryo-EM Structure of a Relaxase Reveals the Molecular Basis of DNA Unwinding during Bacterial Conjugation

**DOI:** 10.1016/j.cell.2017.04.010

**Published:** 2017-05-04

**Authors:** Aravindan Ilangovan, Christopher W.M. Kay, Sandro Roier, Hassane El Mkami, Enrico Salvadori, Ellen L. Zechner, Giulia Zanetti, Gabriel Waksman

**Affiliations:** 1 Institute of Structural and Molecular Biology, Birkbeck, Malet Street, London WC1E 7HX, UK; 2 Institute of Structural and Molecular Biology, University College London, Gower Street, London WC1E 6BT, UK; 3 London Centre for Nanotechnology, University College London, 17–19 Gordon Street, London WC1H 0AH, UK; 4 Institute of Molecular Biosciences, University of Graz, BioTechMed-Graz, Humboldtstrasse 50, 8010 Graz, Austria; 5 School of Physics and Astronomy, University of St Andrews, St Andrews KY16 9SS, UK

**Keywords:** relaxase, TraI, bacterial conjugation, cryo-electron microscopy, type IV secretion system, structural biology

## Abstract

Relaxases play essential roles in conjugation, the main process by which bacteria exchange genetic material, notably antibiotic resistance genes. They are bifunctional enzymes containing a trans-esterase activity, which is responsible for nicking the DNA strand to be transferred and for covalent attachment to the resulting 5′-phosphate end, and a helicase activity, which is responsible for unwinding the DNA while it is being transported to a recipient cell. Here we show that these two activities are carried out by two conformers that can both load simultaneously on the origin of transfer DNA. We solve the structure of one of these conformers by cryo electron microscopy to near-atomic resolution, elucidating the molecular basis of helicase function by relaxases and revealing insights into the mechanistic events taking place in the cell prior to substrate transport during conjugation.

## Highlights

• Relaxases process and transfer plasmid DNAs during conjugation
• The TraI relaxase binds the origin of transfer DNA as a dimer
• TraI can adopt a closed and open conformation, and both co-exist in the dimer
• The presented ssDNA-bound TraI structure reveals the closed “helicase” conformation

## Graphical Abstract

**Figure:**
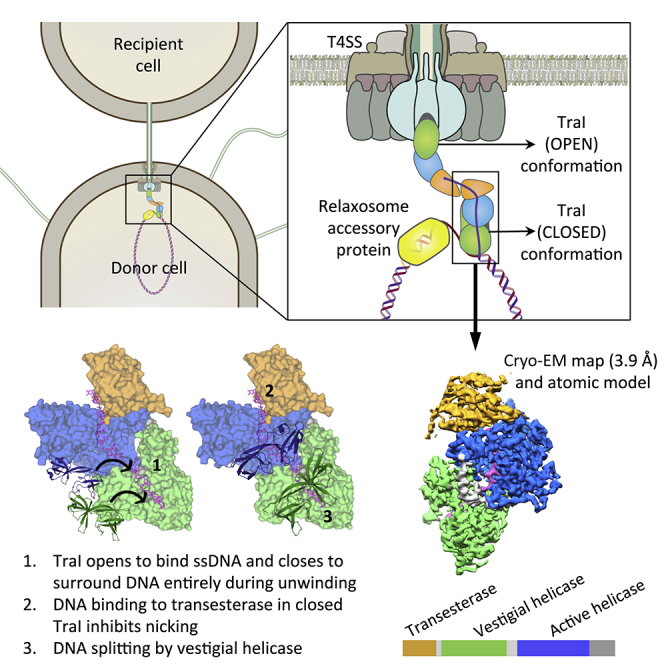

## Teaser

To achieve genetic exchange during bacterial conjugation, two relaxase monomers collaborate, adopting distinct structural conformations to provide the two necessary enzymatic activities for processing the DNA.

## Introduction

Horizontal gene transfer (HGT) is the main process by which bacteria exchange genetic material and thus plays crucial roles in bacterial adaptation and evolution. It is also one of the principal means by which antibiotic resistance genes spread among bacterial pathogen populations (von Wintersdorff et al., 2016). HGT is, for the most part, mediated by conjugative type IV secretion (T4S) systems, versatile transport machineries capable of transporting DNAs and proteins from one bacterium to another and also to eukaryotic hosts.

Conjugative transfer of DNAs among bacteria has been investigated for many years, but structural and mechanistic insights into this process have emerged only recently. Conjugative T4S systems in Gram-negative bacteria are composed of 12 components, termed VirB1–11 and VirD4, that form a large, multi-megadalton complex spanning the double membrane (Ilangovan et al., 2015). This large complex recruits its substrate, single-stranded DNA (ssDNA), and translocates it into a recipient cell via an extracellular pilus (Costa et al., 2016). Substrate recruitment is, however, preceded by a DNA-processing step mediated by another multi-protein complex, termed “the relaxosome” (Ilangovan et al., 2015). For transport of plasmid DNAs, a common substrate of conjugative T4S systems, the relaxosome assembles at a specific sequence on the plasmid DNA, termed “origin of transfer” (*oriT*). The relaxosome is composed of three to four components, its largest and most important being the “relaxase.” The relaxase generally contains at least two conserved domains: (1) a trans-esterase domain responsible for *oriT* recognition, nicking of *oriT* at a particular site termed “*nic*,” and covalent complex formation between a catalytic Tyr residue and the 5′-phosphate end of the nicked (or T-) strand, and (2) a helicase domain responsible for unwinding DNA immediately downstream of *oriT* (Ilangovan et al., 2015). The other relaxosome components are known as “accessory proteins”: these proteins facilitate relaxase recruitment to *oriT* and locally melt *oriT* to enable access to the relaxase. Relaxases are also capable of end-joining in a reverse reaction to the nicking reaction (Ilangovan et al., 2015). End-joining occurs once transport of the nucleo-protein complex is complete.

One of the conjugative systems that have been particularly well studied is that encoded by the F-family plasmids, which include the F, R1 (antibiotic resistance “R” factor obtained from human clinical isolates of pathogenic bacteria), and pED208 plasmids. F-family plasmids encode their own T4S system, as well as their own relaxase and relaxosome components, and thus are self-transmissible plasmids that can mediate their own conjugation to a recipient cell (Lawley et al., 2003). The F plasmid has a special place in the history of science. Indeed, the F plasmid is able to integrate into its *Escherichia coli* host genome and thus conjugate the entire *E. coli* genome into a recipient cell. This discovery made in the 1950s and 1960s signaled the dawn of the field of molecular biology and genetics (Taylor and Thoman, 1964, Wollman et al., 1956).

**Figure 1:**
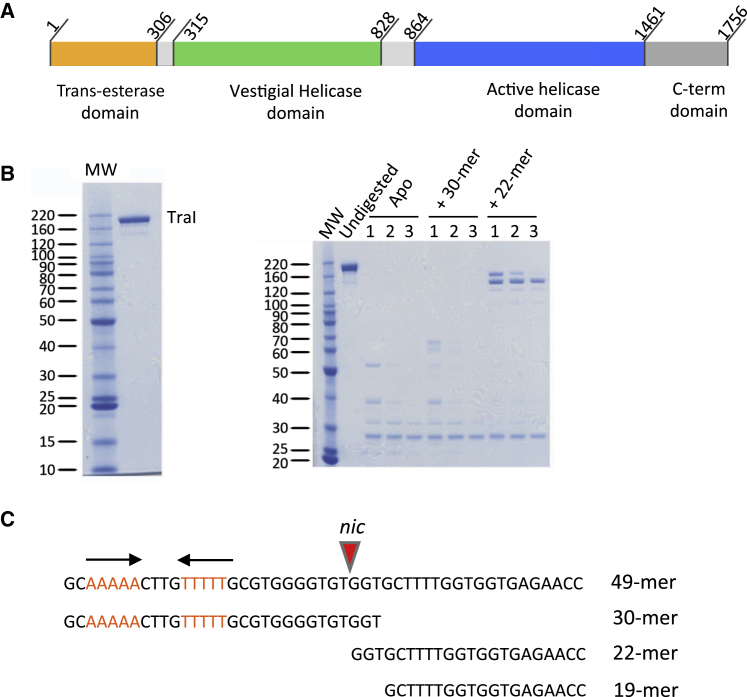
Domain Structure of TraI, Purification of TraI, and Oligonucleotides Used in this Study (A) Primary domain structure of TraI. The four domains of TraI are shown in different colors and labeled accordingly. The linkers between the trans-esterase and vestigial helicase domains and between the vestigial and active helicase domains are colored in light gray. Residue numbering of domain boundaries is derived from the study presented here. (B) Purification of TraI and mild-proteolysis of TraI:ssDNA complexes. Left: TraI purifies as a single band on SDS-PAGE. Right: SDS-PAGE analysis of trypsin-digested TraI bound to various oligonucleotides. TraI alone (Apo) or bound to a 30-mer (+30-mer) or 22-mer (+22-mer) derived from the sequences 5′ or 3′ to the *nic* site, respectively (see sequences in [C]) were digested for 30, 60, or 120 min (lane 1, 2, or 3, respectively) by trypsin and analyzed using SDS-PAGE as described in Method Details. (C) Nucleotide sequences of oligonucleotides used in this study. The 49-mer contains the *nic* site (indicated by a red arrow), 27 nucleotides 5′ to the *nic* site, and 22 nucleotide 3′ to the *nic* site. Red letters and arrows indicate the inverted repeat sequence. The 30-mer, 22-mer, and 19-mer oligonucleotides are shown in register with the 49-mer.

The relaxase of F-family plasmids is TraI and is conserved within the family. It contains four domains (Figure 1A): (1) a trans-esterase domain that executes the nicking and covalent attachment of the T-strand to the relaxase (Datta et al., 2003), (2) a vestigial helicase domain that operates as an ssDNA-binding domain (Dostál and Schildbach, 2010), (3) an active 5′ to 3′ helicase domain, and (4) a C-terminal domain that functions as a recruitment platform for relaxosome components (Guogas et al., 2009, Matson and Ragonese, 2005). Structural investigation of relaxases has focused on individual domains. Notably, the trans-esterase domains of the R388 (Guasch et al., 2003) and F (Datta et al., 2003, Larkin et al., 2005) plasmids have been structurally characterized, and their complexes with *oriT* substrates have been solved, shedding light on the trans-esterification reaction leading to covalent ssDNA-protein complex formation. Other small and incomplete domain structures of F-family TraI have also been solved (Guogas et al., 2009, Redzej et al., 2013, Wright et al., 2012). However, in the absence of a full-length relaxase structure, it is impossible to understand how the various domains cooperate and thus how TraI carries out its function. Here, we report the near-atomic resolution structure of a full-length F-family TraI relaxase, that of the R1 plasmid, determined by single-particle cryo electron microscopy (cryo-EM). This structure was solved with a 22-mer *oriT* T-strand DNA and represents TraI in its “helicase” mode.

## Results and Discussion

### Open and Closed States of TraI

**Figure S1:**
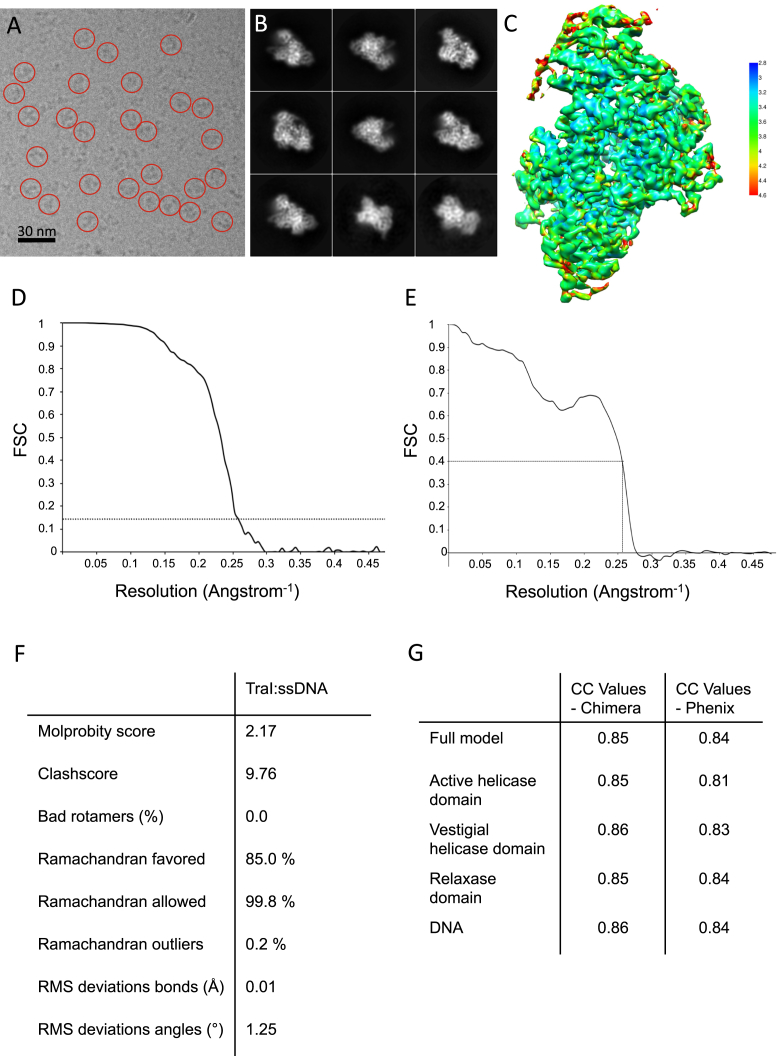
Cryo Electron Microscopy of the TraI:ssDNA Complex, Related to Figure 3 (A) Electron micrograph of the TraI:22-mer ssDNA complex, with some particles highlighted in red circles. The scale bar represents 30 nm. (B) Representative class averages obtained using RELION. These were obtained from the 184,000 particle dataset used to generate the final map. (C) Electron density map visualized at 6.5 σ level and colored according to local resolution (color key in Å on right side of panel). Local resolution was assessed with Resmap (Kucukelbir et al., 2014). The output indicated a resolution range between 3.1 and 10 Å. (D) Resolution of TraI:ssDNA complex as derived from Fourier Shell Correlation (FSC) between independently refined half-maps. The curve represents the mask-corrected FSC between two independently refined half-maps after post-processing in RELION 2.0 (see METHODS), and indicates a resolution of 3.9 Å at FSC = 0.143. (E) FSC between model-derived map and experimental map. The curve represents the FSC between the map derived from the model and the post-processed experimental map, calculated within the same mask used for post-processing in RELION (see METHODS), and indicates that the model correlates very well with the map at the reported resolution of 3.9 Å (FSC at 3.9 Å is equal to 0.396). (F) Model statistics from Molprobity. (G) Cross correlation values between models of the various domains and the experimental map. Correlation coefficients between map and models were calculated using CHIMERA or PHENIX at a resolution of 3.9 Å.

Purified TraI (Figure 1B, left) on its own is prone to aggregation and thus does not crystallize, nor does it produce particles analyzable by cryo-EM (Figure S1). This led us to examine the behavior of TraI in a complex with a number of ssDNAs derived from the sequence of *oriT*, its natural substrate (see Figure 1C for oligonucleotides used in this study). *OriT* encompasses a region of about 350 base pairs, the core of which (exemplified here by the 49-mer oligonucleotide used in this study [Figure 1C]) is the substrate for the relaxase. This 49-mer ssDNA contains three parts: (1) the *nic* site; (2) a region of 27 nucleotides, 5′ to this site, that includes an inverted repeat sequence (in red and also indicated by arrows in Figure 1C); and (3) a 22-nucleotides region (22-mer), 3′ to the *nic* site (Figure 1C). Mild proteolysis was used to probe the conformations of TraI resulting from the binding of ssDNA. This technique enables conformational species to be rapidly probed and identified. We observed two distinct behaviors in solution depending on the oligonucleotides employed. When bound to a 30-mer oligonucleotide containing the 5′ inverted repeat sequence and the *nic* site (Figure 1C; this 30-mer oligonucleotide is known to interact with the trans-esterase domain and not the helicase domains), TraI is susceptible to rapid degradation by mild trypsin proteolysis (Figure 1B, right). In contrast, when bound to a 22-mer oligonucleotide derived from the sequence 3′ to the *oriT*’s *nic* site and thus a substrate for the helicase domains (Figure 1C), the complex becomes more resistant to trypsin (Figure 1B, right). Thus, depending on the oligonucleotide bound, TraI exhibits two conformations: one, observed when the ssDNA is bound to the trans-esterase domain, is open and thus accessible to proteolytic cleavage, while the other, observed when ssDNA is bound to the helicase domains, is closed and thus less accessible to protease degradation.

### Two TraI Molecules Load on Each Side of the *Nic* Site

**Figure 2:**
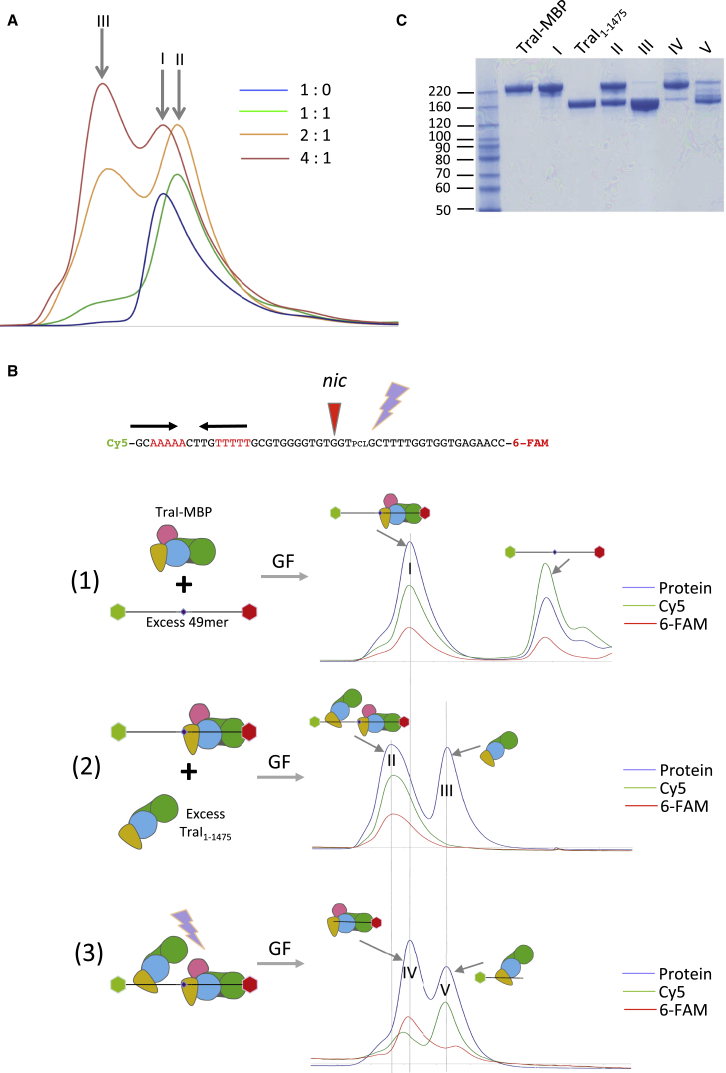
Recruitment of a TraI Dimer on *OriT* (A) Gel filtration (GF) of TraI and TraI:49-mer ssDNA complexes. TraI alone (blue) and various mixtures of TraI and 49-mer ssDNA (1:1, green; 2:1, orange; 4:1, red) were analyzed using GF as described in Method Details. Three peaks were observed: peak I, corresponding to Apo TraI; peak II, corresponding to a 1:1 mixture of TraI:ssDNA (migrating slightly more rapidly than the Apo form of TraI as it is in a closed conformation, while Apo TraI is in an open conformation [see Figure 1]); and peak III, corresponding to two molecules of TraI bound to the 49-mer. Note that in the 4:1 TraI:ssDNA experiment, excess Apo TraI is observed, which is expected. This panel only reports on the OD_280_ absorbance. For more complete data including the OD_260_ absorbance monitoring DNA, see Figure S2A. (B) GF analysis of various TraI:49-mer_Cy5∗6-FAM_ complexes. Top: The ssDNA sequence describes the 49-mer_Cy5∗6-FAM_ used. The *nic* site is shown as well as the position of the Cy5, 6-FAM, and the photocleavable (PCL) site. Below: We show the results of a series of successive experiments: panels on the left illustrate the various molecules in reaction mixtures analyzed using GF, the profiles of which (blue, red, and green lines reporting on the absorption at 280 [protein], 497 [6-FAM], and 649 [Cy5] nm, respectively) are shown immediately to the right. In these illustrations, the 49-mer_Cy5∗6-FAM_ oligonucleotide is shown as a line, ending with hexagons representing the Cy5 (green) and 6-FAM (red) labels; the PCL site is indicated as a small black prism. TraI is depicted as a four-colored object—orange, green, blue, and gray—schematically representing the four domains of TraI: the trans-esterase, vestigial helicase, active helicase, and C-terminal domains, respectively. The pink hexagon represents MBP in the TraI-MBP fusion. In a first step, a 1:1 complex of TraI-MBP bound to 49-mer_Cy5∗6-FAM_ is purified using GF resulting in a protein:ssDNA peak, peak I, and a second peak with excess unbound 49-mer_Cy5∗6-FAM_. In a second step, an excess of TraI_1-1475_ (represented as TraI except for the C-terminal domain, which is absent) is added to the 1:1 TraI-MBP:49-mer_Cy5∗6-FAM_ complex, and the resulting TraI-MBP:49-mer_Cy5∗6-FAM_:TraI_1-1475_ complex is purified by GF (corresponding to peak II; peak III contains the excess TraI_1-1475_). In step three, the purified TraI-MBP:49-mer_Cy5∗6-FAM_:TraI_1-1475_ complex is irradiated at 365 nm as described in Method Details, and the mixture analyzed by GF. Two peaks are observed: peak IV containing primarily TraI-MBP bound to 6-FAM-containing fluorescent ssDNA and peak V containing primarily TraI_1-1475_ bound to Cy5-containing fluorescent ssDNA. (C) SDS-PAGE analysis of TraI-MBP, TraI_1-1475_, and peaks I, II, III, IV, and V (see [B]). See also Figure S2.

**Figure S2:**
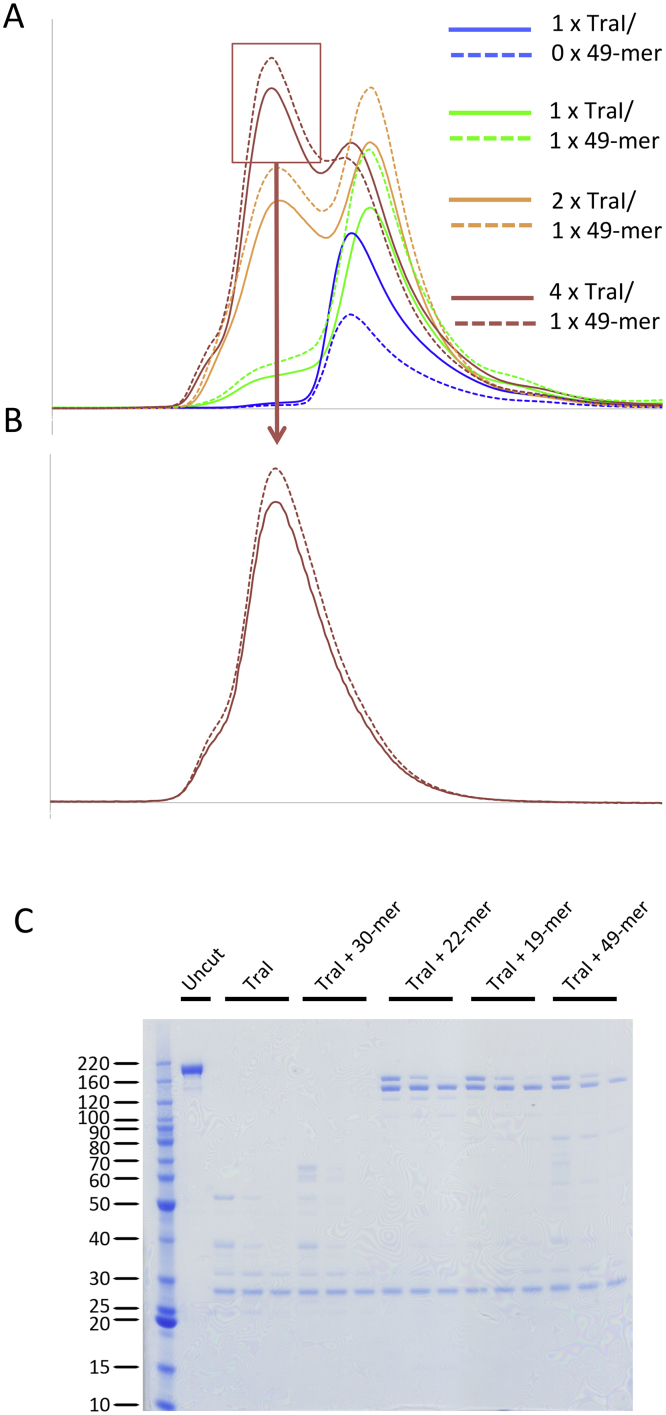
Formation and Purification of the TraI:49-mer ssDNA Complex, Related to Figures 1 and 2 (A) Same as Figure 2A but showing in addition the OD_260_ signal in dotted lines. (B) Purification of the 2:1 TraI:49-mer complex. The peak indicated in Figure S2A with a red rectangle was re-loaded onto a gel filtration column for the purpose of further purifying the 2:1 TraI:49-mer. (C) Same as in Figure 1B, right panel but showing in addition the mild-proteolysis of the 1:1 TraI:19-mer and 1:1 TraI:49-mer complexes.

We next engaged in a series of biochemical experiments aimed at examining the functional relevance of our discovery of an open and closed conformer of TraI. We first asked whether the two TraI conformers can co-exist on *oriT*; i.e., can they form an *oriT*-mediated dimer. We showed that, indeed, a TraI dimer can be formed on the 49-mer oligonucleotide described above (see details in legends to Figures 2A and S2). This dimer is stable as it can be purified to homogeneity (Figure S2B). We then investigated whether this dimer of TraI contains TraI molecules bound on either side of the *nic* site. To test this, we used a 49-mer oligonucleotide similar to the one used previously, but for the presence of a photo-activable cleavage site located three bases downstream to the *nic* site, and two different fluorophores at either ends, cyanine 5 (Cy5) at the 5′ end and 6-carboxyfluorescein (6-FAM) at the 3′ end (this oligonucleotide is termed 49-mer_Cy5∗6-FAM_ [top of Figure 2B]). Photo-cleaving at 365 nm wavelength would result in two oligonucleotides: the 30-mer described previously and a 19-mer (see sequence in Figure 1C), each singly labeled with Cy5 and 6-FAM, respectively. We also used two constructs of TraI: a full-length TraI fused to MBP at the C terminus (TraI-MBP) and a shorter version of TraI (residues 1 to 1475; TraI_1-1475_) where the C-terminal domain was deleted. The C-terminal domain of TraI does not interfere with trans-esterase and helicase activities, and the difference in molecular weight between TraI-MPB (240 kDa) and TraI_1-1475_ (155 kDa) is sufficient to separate these two species by gel filtration. The dimer was obtained by forming and purifying first a 1:1 TraI-MPB:49-mer_Cy5∗6-FAM_ complex and then adding excess TraI_1-1475_ and purifying the 1:1:1 TraI-MPB:49-mer_Cy5∗6-FAM_:TraI_1-1475_ complex (see Method Details). This complex was subjected to photo-cleavage, and the products of the cleavage reaction were analyzed using gel filtration (Figures 2B and 2C). Two peaks were observed: a higher molecular weight peak containing TraI-MBP primarily bound to the 6-FAM-labeled 19-mer and a lower molecular weight peak containing TraI_1-1475_ primarily bound to the Cy5-labeled 30-mer. Moreover, mild trypsin proteolysis of 1:1 complexes between full-length TraI and an unlabelled 49-mer or a 19-mer (with a sequence identical to that of the singly 6-FAM-labeled 19-mer released by photo-cleavage of the 49-mer_Cy5∗6-FAM_ oligonucleotide) resulted in band patterns identical to those obtained for the TraI/22-mer complex (Figure S2C). We conclude that (1) TraI dimer formation is entirely mediated by ssDNA and (2) *oriT* can accommodate two TraI monomers, one loading onto the 3′ side of the *nic* site and adopting a closed conformation, the other loading onto the 5′ side of that site and adopting an open conformation.

### Structure Determination of the Closed State of TraI

**Figure 3:**
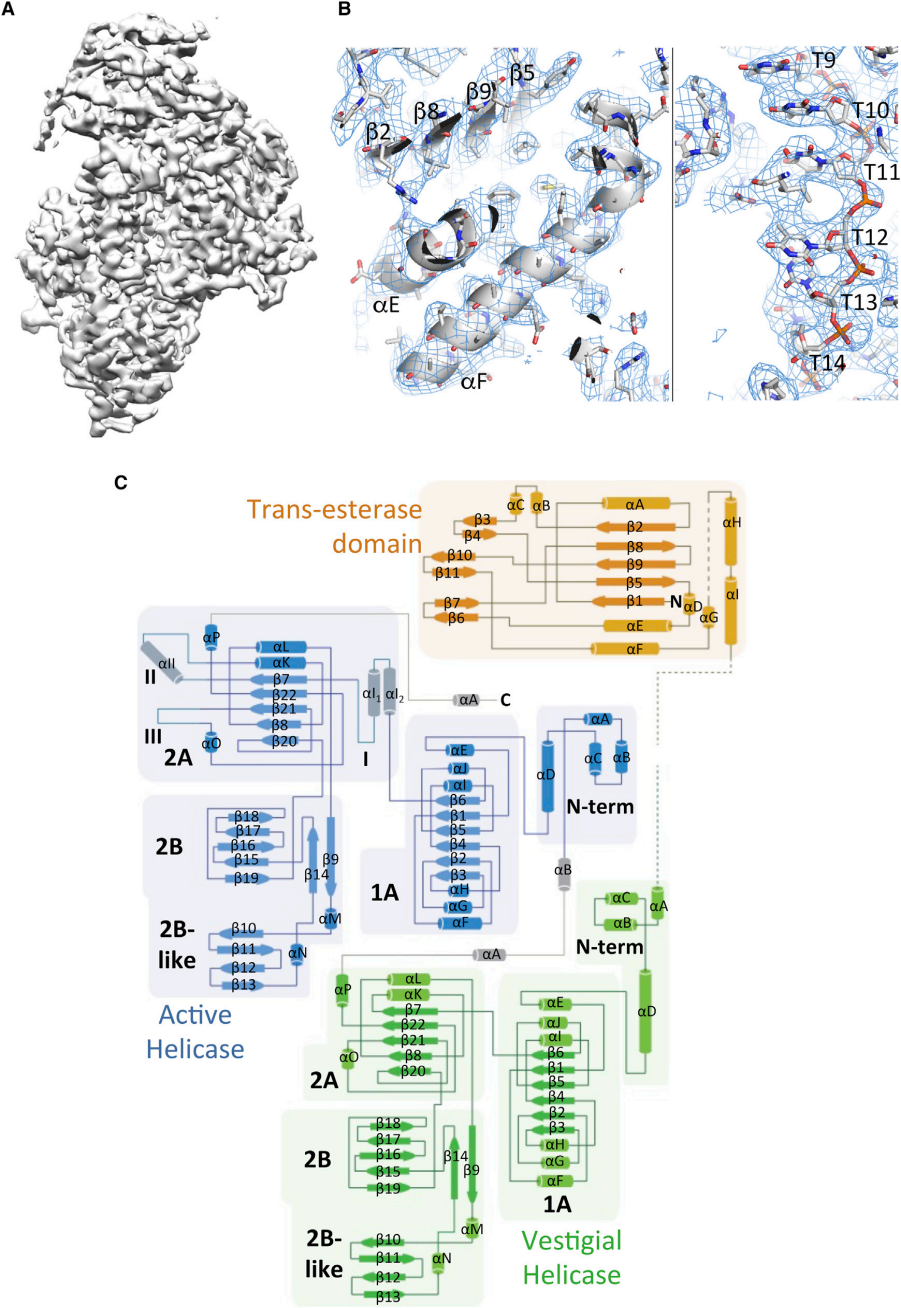
Representative Regions of the Cryo-EM Map and Topology Diagram of TraI Bound to the 22-mer Oligonucleotide (A) Overview of the 3.9 Å electron density, contoured at 6.5 σ level. (B) Two representative regions of the electron density. Electron density map contoured at a 5 σ level is shown in chicken-wire representation, color-coded in gray-blue. The final model built into the map is shown in a ribbon-and-stick representation color-coded blue, red, orange, and gray for nitrogen, oxygen, phosphorus, and carbon atoms, respectively. Secondary structures are labeled as well as nucleotides. The regions depicted are in the trans-esterase domain (left) and in the ssDNA (right). (C) Topology diagram of the TraI:22-mer ssDNA complex. The trans-esterase, vestigial helicase, and active helicase domains are shown in orange, green, and blue, respectively. Helices and strands are shown as rectangles and block arrows, respectively. Secondary structures in the active helicase domain that are not found in the vestigial helicase domain are shown in lighter blue and are labeled regions I, II, and III as in Figure 5A. The 2B and 2B-like parts of the 2B/2B-like sub-domains are indicated. See also Figure S1.

While the open form of TraI formed aggregated samples unsuitable for cryo-EM analysis, the closed form of TraI bound to the 22-mer oligonucleotide could be readily vitrified on grids and formed well-dispersed particles amenable to cryo-EM data collection and single-particle reconstruction (Figure S1A). Two datasets were collected: the first on the 22-mer ssDNA-bound TraI resulted in a map with an average resolution of 4.5 Å, while the other on the 22-mer ssDNA-bound TraI vitrified in the presence of 1 mM AMP-PNP yielded a map with an average resolution of 3.9 Å. No density for AMP-PNP was observed near the ATP-binding site, indicating that AMP-PNP had not bound. Thus, increased resolution was not due to the presence of AMP-PNP but rather improved conditions during data collection (see Method Details), and, therefore, we used this higher resolution map to build a model of the TraI:22-mer complex (Figures 3A and S1B–S1D). In this map, all secondary structures were clearly visible, as well as most side chains (Figure 3B), and a model for the trans-esterase, vestigial helicase, and active helicase domains could be readily built and refined with excellent stereochemistry (Figures S1E and S1F). Density for the ssDNA was clearly visible for both the ribo-phosphate backbone and the bases (Figure 3B). However, density for the bases was uniformly shaped, and no difference was observed between purines and pyrimidines, suggesting that ssDNA might not bind TraI in a single register, which is expected given that helicases must be able to slide along the ssDNA and thus must minimize sequence binding specificity. Also, only 18 out of the 22 nucleotides were visible in the electron density, and thus, a 18-mer poly-T, arbitrarily numbered from 5 to 22, was built.

### General Architecture of the TraI:ssDNA Complex

**Figure 4:**
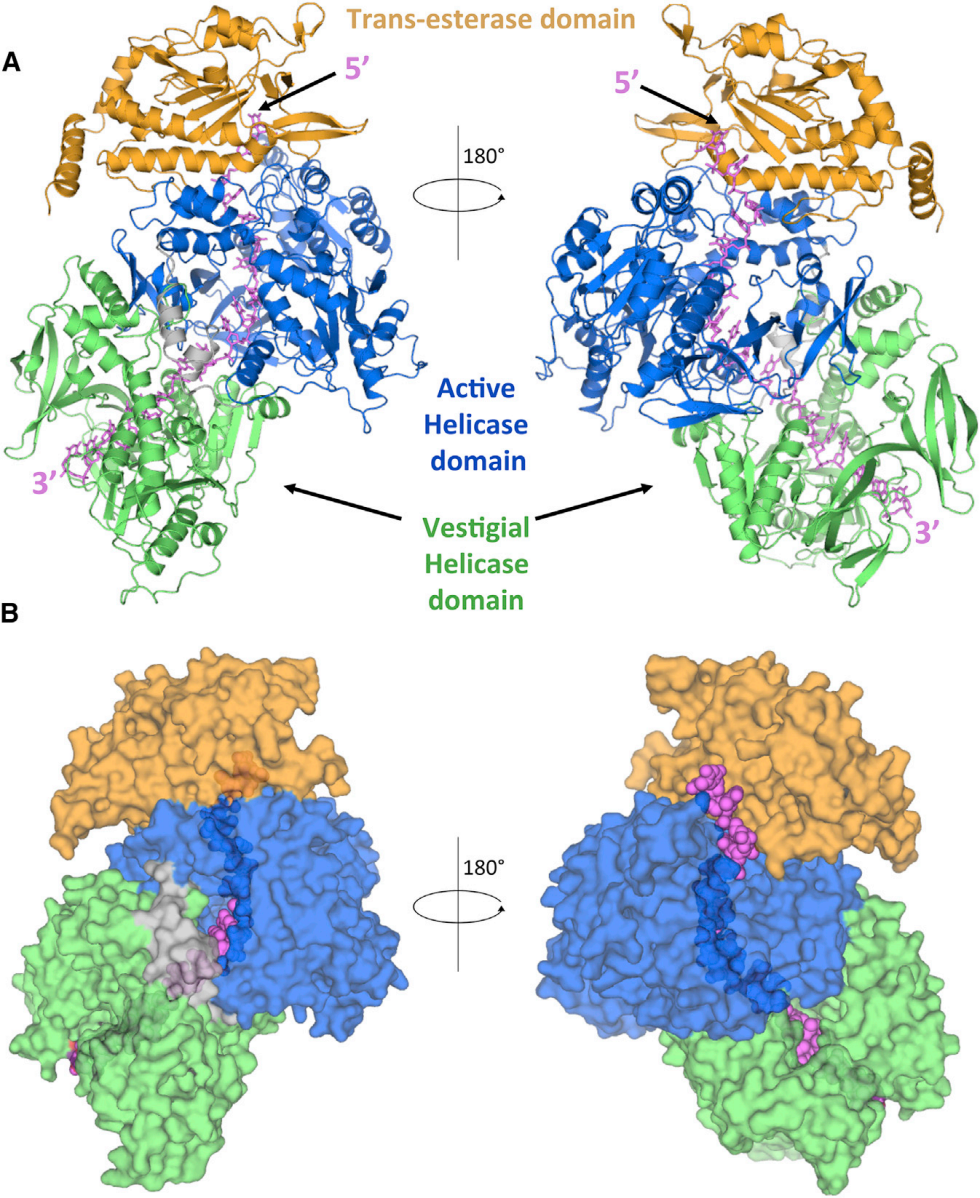
Structure of the TraI:ssDNA Complex (A) Overall structure of the TraI:ssDNA complex. The protein is shown in ribbon representation color-coded orange, green, and blue for the trans-esterase, vestigial helicase, and active helicase domains, respectively, while the DNA is shown in stick representation color-coded in magenta. The linker region between the vestigial and active helicase domains is shown in light gray. The left and right panels show the structure 180° apart along the vertical axis. (B) Surface diagram of TraI:ssDNA complex. The protein and the ssDNA are shown in semi-transparent surface and ball representation, respectively. Color-coding and orientation are as in (A). See also Figures S5 and S6.

The primary sequence of TraI contains three linearly arranged domains: a trans-esterase domain, a vestigial helicase domain, and an active helicase domain, indicated in orange, green, and blue, respectively, in Figure 1A. However, in the three-dimensional structure, it is the active helicase domain that is closer to the trans-esterase domain, while the vestigial helicase domain locates at the opposite end of the structure (Figures 3A, 3C and 4A). A long linker sequence between the C terminus of the trans-esterase domain (residue 298) and the N terminus of the vestigial helicase domain (residue 314) bridges the distance between these two domains. Part of this linker is disordered (residues 307–314) as no electron density is observed for these residues. Following the vestigial helicase domain, another long linker between residues 828 and 864 meanders to connect the vestigial helicase domain to the active helicase domain. As indicated by its well-resolved electron density, this linker is completely structured. Electron density ends at residue 1473, beyond which 283 residues form a C-terminal domain, the structure of which is partly known in isolation (residues 1476–1628) (Guogas et al., 2009), but could not be traced here, suggesting that it is flexibly located. This domain is thought to provide a platform for the assembly of other components of the relaxosome (Ragonese et al., 2007). These proteins might be required to immobilize the TraI C-terminal domain in an ordered conformation.

**Figure S3:**
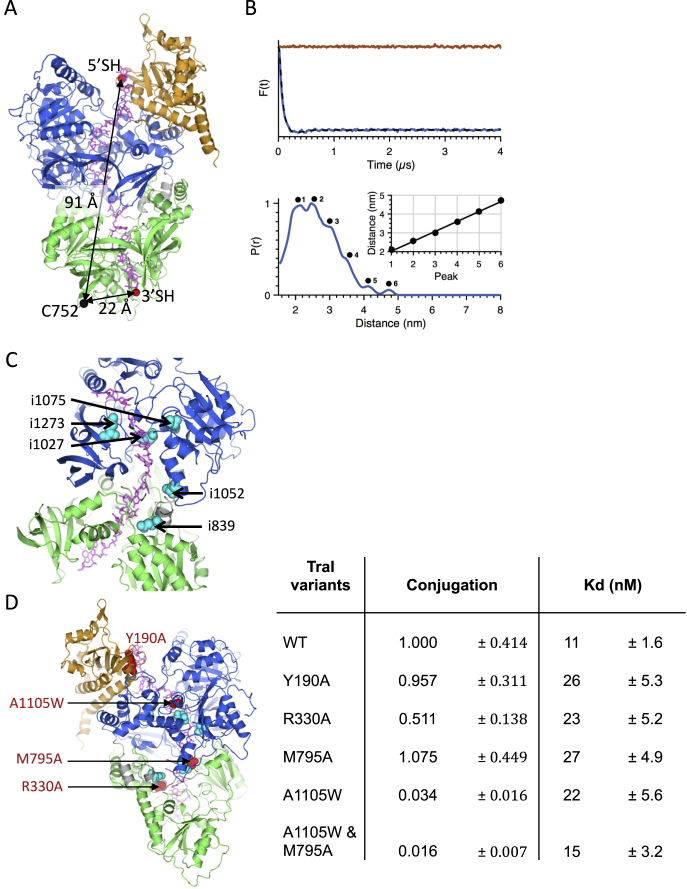
Validation of the Structure, Related to Figure 4 (A) Distance measured in the TraI:22-mer ssDNA structure between residue 752 (Cα atom) and either the 3′ or 5′ end of the 22-mer ssDNA. The structure is shown as in Figure 4. The 3′ or 5′ ends of the ssDNA and C752 are labeled. (B) EPR distance measurement confirming the polarity of the 22-mer ssDNA bound to TraI. Top panel: dipolar evolution following baseline correction measured on TraI_R752C_:22-mer_3′SH_ complex (blue line) gives a clear response indicative of a short mean distance, whereas TraI_R752C_:22-mer_5′SH_ complex (red line) is flat indicative of a distance beyond the range of detection. Bottom panel: distance distribution for TraI_R752C_:22-mer_3′SH_ complex from the data reported in the top panel. A series of peaks are observed in the distance distribution, labeled 1 to 6. The inset depicts distance versus peak number, showing that the peaks are equidistant with a step of 5.3 Å, suggesting flexibility in the register of the ssDNA relative to the protein. (C) Mutational study by Haft et al. (2006). 31-residues insertions that disrupt conjugation were introduced at positions indicated in the structural model. These map to the ssDNA-binding site as determined by the structure of TraI bound to ssDNA presented here. (D) Mutational analysis of the ssDNA-binding site. Four single-site mutations and one double-site mutation were introduced at positions indicated in the panel at left: Y190A in the trans-esterase domain, R330A and M795A in the vestigial helicase domain, A1105W in the active helicase domain, and A1105W/M795A double mutant. The affinity of the resulting protein variants for ssDNA was tested as well as their effect on conjugation, the transfer of ssDNA from a donor cell to a recipient cell. The location of these residues is shown in the left panel, and the dissociation constants and conjugation frequencies (normalized to wild-type (WT)) are reported in the right panel. The interface between ssDNA and TraI being large (2608 Å^2^), it is expected that the effect of most single residue mutations on ssDNA-binding would be small. Also, ssDNA translocases must constantly process ssDNA along their ssDNA-binding interface and thus residue-specific “local” binding must remain weak as, otherwise, they would stall. Remarkably, two- to three-fold decrease in affinity is observed for most mutations we investigated, except for the double A1105W/M795A mutant, which has a higher affinity for ssDNA than the corresponding single mutants from which it was derived. Conjugation efficiencies remain at wild-type levels for Y190A and M795A, showing that affinity would need to be further decreased before an effect on conjugation is observed. A1105W has a dramatic effect on conjugation, presumably because its Trp residue might jam the helicase activity of the protein. Remarkably, adding M791A to A1105W potentiates the impairment of conjugation observed for A1105W alone. Thus, mutating residues in the observed interface between TraI and ssDNA affects binding and some of these mutations have dramatic biological effects, providing further validation of the structure. All results are from at least three independent experiments.

The ssDNA traverses longitudinally the entire structure of TraI, with its 5′ half bound to the trans-esterase and the active helicase domains, while its 3′ half is observed bound to the vestigial helicase domain (Figures 4A and 4B). This orientation of the ssDNA was confirmed using electron paramagnetic resonance (EPR) spectroscopy, a method particularly well suited to accurately determine distances between two paramagnetic labels by measuring their dipolar coupling (Pannier et al., 2000). In this experiment, residue 752 at the base of the vestigial domain (see position in Figure S3A) was mutated to Cys, and the purified TraI_R752C_ protein was bound to 22-mer ssDNAs thiolated at either the 3′ end or the 5′ end (referred to as 22-mer_3′SH_ or 22-mer_5′SH_), resulting in the formation of either a TraI_R752C_:22-mer_3′SH_ or a TraI_R752C_:22-mer_5′SH_ complex. These two complexes were then reacted with the spin-label PROXYL using methanethiosulfonate chemistry as described in Method Details. TraI does not contain any cysteine residues, and thus, Cys752 is the only residue in TraI_R752C_ that can be labeled. Successful double-labeling of both complexes was monitored using continuous-wave EPR (data not shown). Next, distance measurements between labeled Cys752 and labeled 3′SH or labeled 5′SH were carried out using double electron-electron resonance (DEER) spectroscopy. Residue 752 was chosen as an appropriate position for labeling because, in the structure, it is 22 Å away from the 22-mer’s 3′ end and 91 Å distant from the 22-mer 5′ end. 22 Å is an ideal distance for DEER measurements while 91 Å is beyond the limit of detection. Thus, we would expect to see a clear signal for the doubly labeled TraI_R752C_:22-mer_3′SH_ complex and no signal for the TraI_R752C_:22-mer_5′SH_ complex. And, indeed, a DEER signal is observed with the 22-mer_3′SH_ and not with the 22-mer_5′SH_ (Figure S3B, top). This result unambiguously demonstrates that the polarity of the ssDNA as modeled in the structure is correct. The distance distribution between spin labels was next derived for the doubly labeled TraI_R752C_:22-mer_3′SH_ complex (Figure S3B, bottom). Interestingly, the distance distribution is not a single peak (as would be expected for a single discrete distance) or a smooth function (as would be expected for a broad distribution of distances), rather the distribution suggests a series of regularly spaced overlapping peaks, 5.3 Å apart, with the first (and highest) centered at 21 Å, which is close to the distance observed in our structural model between residue 752 and the ssDNA 22-mer’s 3′ end. We interpret the additional, regularly spaced peaks as indicative of different ssDNA registers relative to position 752. This result is consistent with the previous suggestion (see above) that the register of the ssDNA along the ssDNA-binding surface might not be strictly defined.

### Structures of the TraI Domains

**Figure S4:**
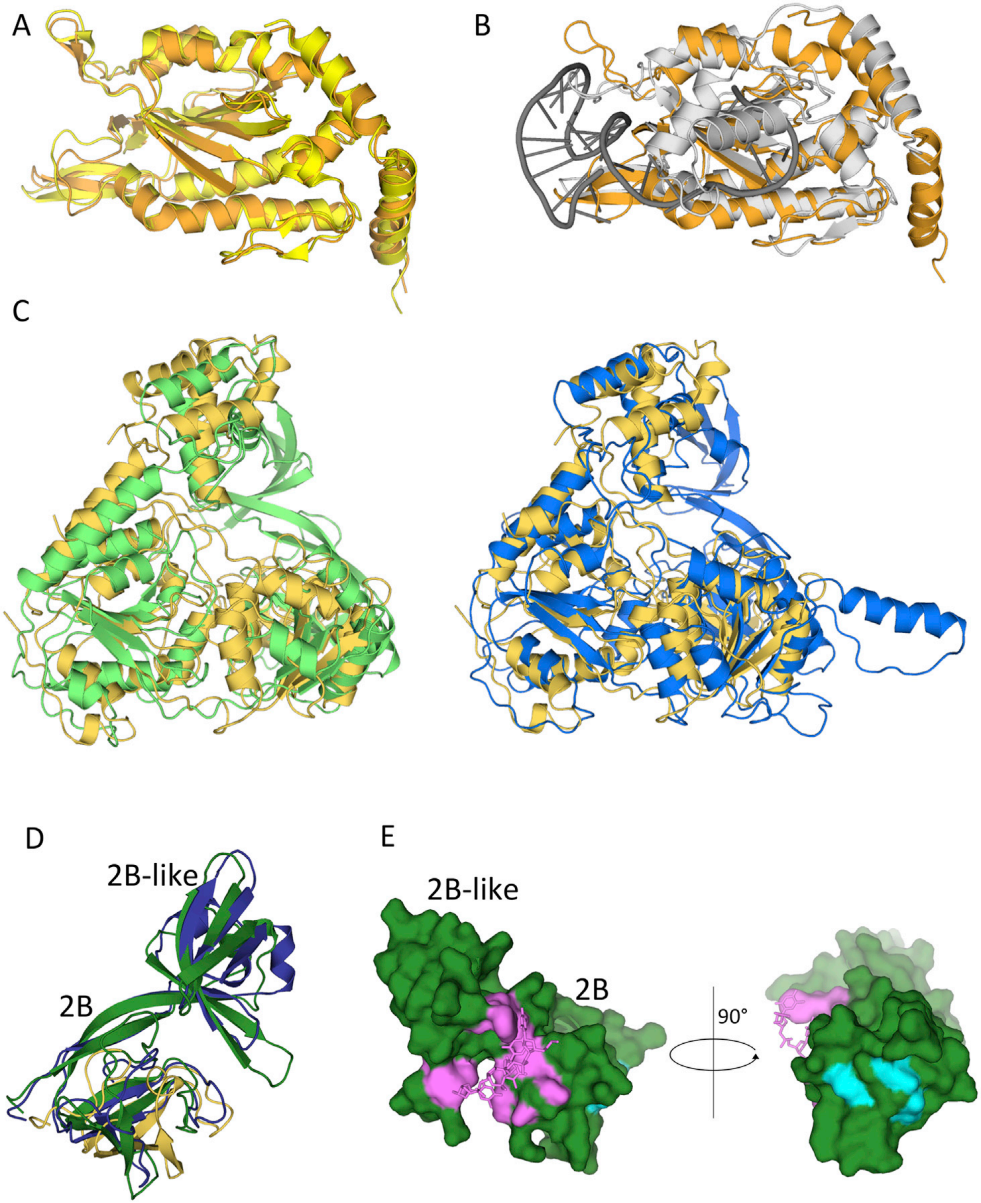
Superposition of TraI Domains with Domains of Known Helicase and Trans-esterase Domain Structures, Related to Figures 6 and 7 (A) Superposition of the structure of the trans-esterase domain of TraI from the structure of the full-length TraI presented here (in orange) with the structure of the TraI trans-esterase domain crystallized in isolation (in yellow). Both structures are in ribbon representation. (B) Superposition of the structure of the trans-esterase domain of TraI from the structure of the full-length TraI presented here (in orange) with the structure of the trans-esterase domain of TrwC (in gray) crystallized in isolation with a ssDNA (in dark gray). All structures including the ssDNA are in ribbon representation. (C) Superposition of the structures of the TraI helicase domains with RecD2. Left: superposition of the structure of the vestigial helicase domain of TraI (in green) with RecD2 (in yellow). Right: superposition of the structure of the active helicase domain of TraI (in blue) with RecD2 (in yellow). All structures are in ribbon representation. (D) Superposition of the 2B/2B-like sub-domains of the vestigial (dark green) and active (dark blue) helicase domains with RecD2 (yellow). The two 2B/2B-like sub-domains of TraI have essentially similar structures but are larger than the 2B sub-domain of RecD2, containing one additional domain that was termed “2B-like” (Redzej et al., 2013) because of its structural similarity with the 2B domain of RecD2 (see main text). As shown in Figures 7A and 7B, ssDNA binds between the 2B and 2B-like sub-domains. (E) Surfaces of the 2B/2B-like sub-domain of the vestigial helicase domain involved in ssDNA binding (in magenta) and in recruitment to the T4S system (in cyan). The structure of the protein is in surface representation while that of the DNA is shown in stick representation (also in magenta). Left panel: the orientation is 180° away along the vertical axis from the orientation shown in panel D. Right panel: the orientation is 90° away clockwise along the vertical axis from the orientation shown at left.

The structure of the trans-esterase domain of TraI is known in isolation and superimposes very well with the equivalent domain in the structure presented here (root-mean-square deviation [RMSD] in Cα atoms of 1.3 Å; Figure S4A). It has been described as a “prolate ellipsoid” with a central β sheet flanked by two α helices on each side (Datta et al., 2003). The trans-esterase domain also superimposes well with the equivalent domain of another relaxase, TrwC, encoded by the plasmid R388 (also determined in isolation; Figure S4B) (Guasch et al., 2003).

**Figure 5:**
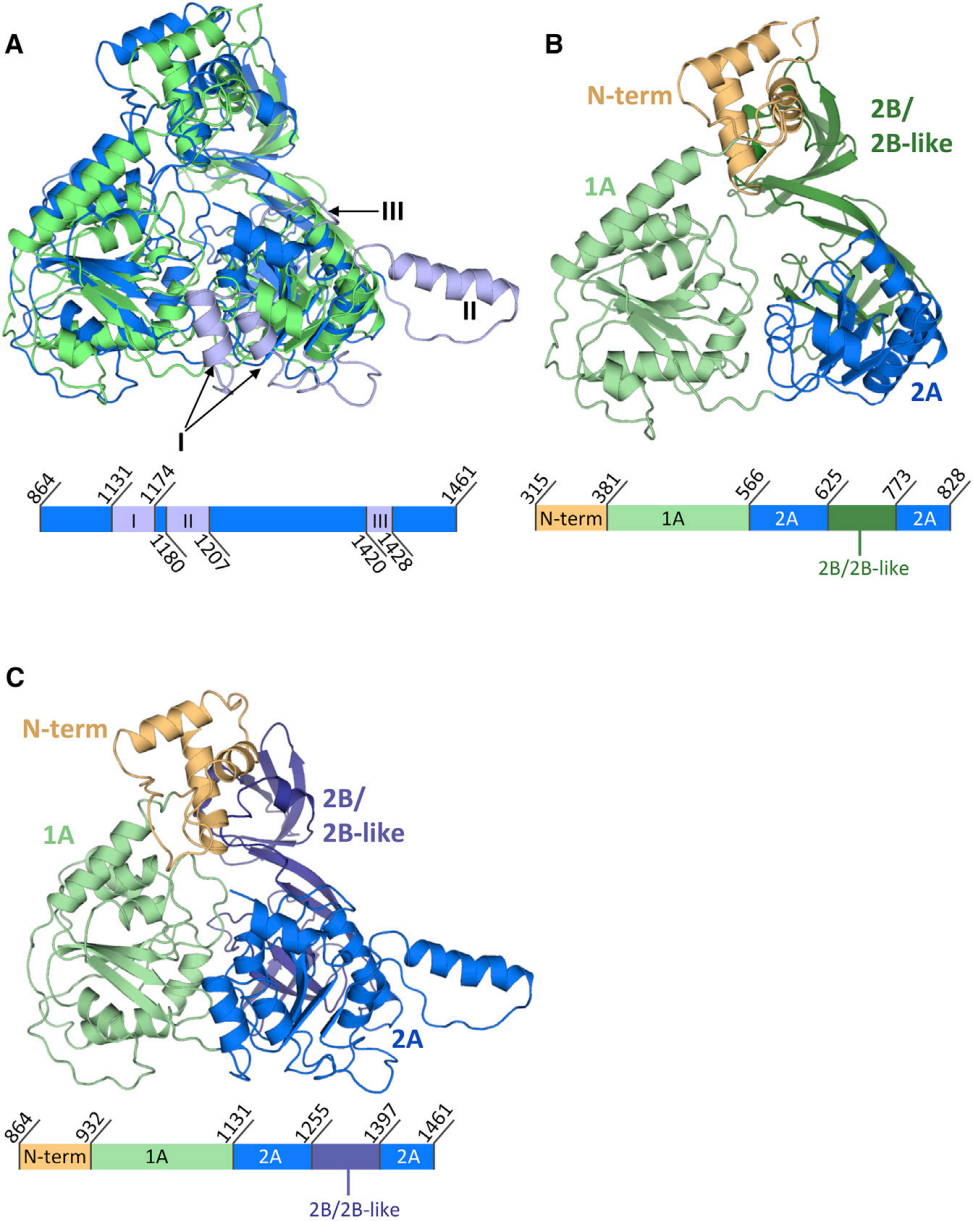
Sub-domain Structures of the Vestigial and Active Helicase Domains (A) Superposition of the structure of the vestigial and active helicase domains. Top: Structural superposition of the vestigial (green) and active (blue) helicase domains. Both are in ribbon representation. Regions found in the active helicase domain but not in the vestigial helicase domain are shown in light blue and labeled I, II, and III as in Figure 3B. Bottom: Schematic representation of the primary sequence of the active helicase domain. Regions I–III are shown in light blue, and their boundary residues are indicated. (B) Structure of the vestigial helicase domain color-coded by sub-domains. Top: Structure of the vestigial helicase domain in ribbon representation with various sub-domains color-coded as in the bottom. Bottom: Schematic representation and sub-domain boundaries of the primary sequence of the vestigial helicase domain with the N-term, 1A, 2A, and 2B/2B-like sub-domains color-coded in light orange, green, blue, and dark green, respectively. (C) Structure of the active helicase domain color-coded by sub-domains. Top: Structure of the active helicase domain in ribbon representation with various sub-domains color-coded as in the bottom. Bottom: Schematic representation and sub-domain boundaries of the primary sequence of the active helicase domain with the N-term, 1A, 2A, and 2B/2B-like sub-domains color-coded in light orange, green, blue, and dark blue, respectively.

The vestigial and active helicase domains of TraI have similar structures, superimposing with an overall RMSD in Cα atoms of 3.8 Å (Figure 5A). They contain four sub-domains, termed N-terminal (N-term), 1A, 2A, and 2B (Figures 3C, 5B, and 5C). Both exhibit the classical helicase sub-domain organization of the SF1A/B family but resemble most that of the RecD2 helicase from *Deinococcus radiodurans*, an archetypal SF1B family helicase that exhibits the same 5′ to 3′ directionality as TraI (Saikrishnan et al., 2009). RecD2 also contains four sub-domains, termed similarly, and these domains superimpose well with the corresponding TraI domains, with a RMSD in Cα position of 5.1 Å between RecD2 and the vestigial helicase TraI domain and 3.3 Å between RecD2 and the active helicase TraI domain (Figure S4C). The N-terminal domain forms an α-helical bundle while the two following domains, 1A and 2A, both exhibit a RecA-like fold (Figure 3C). However, the 2B sub-domains of TraI differ substantially from that of RecD2. The 2B sub-domains in TraI are formed by residues 625–773 in the vestigial helicase domain and residues 1255–1397 in the active helicase domain (Figures 3C, 5B, and 5C). Both are sequences inserted within the 2A sub-domains; i.e., sequences before and after the 2B sub-domains rejoin to form the 2A sub-domains. This is also the case for the 2B sub-domain of RecD2. However, in TraI, the 2B sub-domains are much larger, containing additional sequences that themselves form an additional domain (Figure S4D). In a prior publication describing the structure of the vestigial helicase TraI 2B sub-domain in isolation, we named this additional domain “2B-like” because of its structural similarity with the 2B domain of RecD2 (Redzej et al., 2013). Thus, in the study presented here, we will thereafter refer to the 625–773 region in the vestigial helicase domain and the 1255–1397 region in the active helicase domain as “2B/2B-like sub-domains.”

The 1A sub-domains of the vestigial and active helicase domains are very similar and overlap with an RMSD in Cα atoms of 2.9 Å (Figure 5A). This is also the case for the 2B/2B-like sub-domains of the vestigial and active helicase domains: they superpose well with each other with an RMSD of 2.6 Å (Figure S4D). However, there are substantial differences in the 2A sub-domains: while their cores are structurally very similar (RMSD of 2.5 Å), there are loops and secondary structures present in the active helicase domain, but absent in the vestigial helicase domain, between residues 1131–1174, residues 1180–1207, and residues 1420–1428 (indicated as I, II, and III, respectively, in Figures 3C and 5A in light blue). Regions I and III are present in all helicases and are essential parts of the ATP- or ssDNA-binding sites of helicases, respectively. The absence of these regions, together with the absence of the Walker A and B motifs, explains why the vestigial helicase domain no longer functions as a DNA unwinding motor. Region II, however, extends out on the side of and away from the 2A domain, and thus, it is unclear what the role of this sequence insertion is.

### The ssDNA-Binding Site

**Figure 6:**
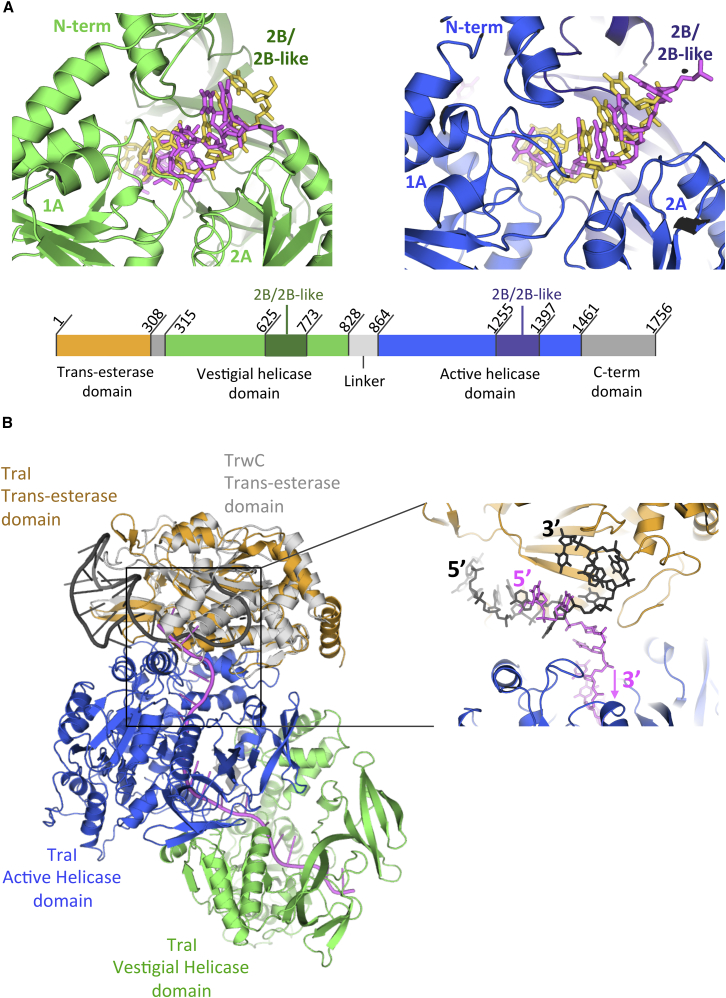
ssDNA-Binding to TraI (A) Comparison of the ssDNA-binding mode of the vestigial and active helicase domains of TraI with that of RecD2. Details of residue-specific interactions with the ssDNA are provided in Figure S5. Top left: The structures of the vestigial helicase domain of TraI and that of RecD2 were aligned as shown in Figure S4C, left. The TraI domain is shown in ribbon representation color-coded in green except for its 2B/2B-like sub-domain, which is in dark green. The portion of the ssDNA bound to this TraI domain (nucleotides 16 to 22) is shown in stick representation color-coded in magenta. The ssDNA bound to RecD2 is shown in stick representation but color-coded in yellow. Top right: Comparison of the ssDNA-binding mode of the active helicase domain of TraI with that of RecD2. Same as in left panel except that the TraI domain used for representation is the active helicase domain; that the structural alignment with RecD2 is as in Figure S4C, right; and that the portion of ssDNA binding to this TraI domain is from nucleotides 9 to 14 of the 22-mer. This TraI domain is shown in blue except for its 2B/2B-like sub-domain, which is in dark blue. Bottom: Schematic representation of the entire TraI sequence mapping the various domains and sub-domains shown in top panels; color-coding is as in the top panels. (B) Structural basis of the negative cooperativity observed between the ssDNA-binding site of the trans-esterase domain and that of the helicase domains. Left: Overall view of the structure of the TraI:22-mer complex superposed with that of the TrwC trans-esterase domain bound to an ssDNA (PDB: 1OMH). TraI is shown in the same orientation, representation, and color-coding as in Figure 4A, right. The TrwC trans-esterase domain is shown as in Figure S4B, color-coded in light gray. The ssDNA bound to the TrwC trans-esterase domain is shown in cartoon representation color-coded in dark gray. The ssDNA bound to TraI is shown in cartoon representation color-coded in magenta. The rectangle indicates the region zoomed into and represented in the right sub-panel. Right: Structural models in the region of the structure comprised within the rectangle shown left. The superposed proteins are as left, but ssDNAs are in stick representation, color-coded dark gray for the ssDNA bound to TrwC and magenta for the ssDNA bound to TraI. See also Figures S4, S5, and S6.

**Figure S5:**
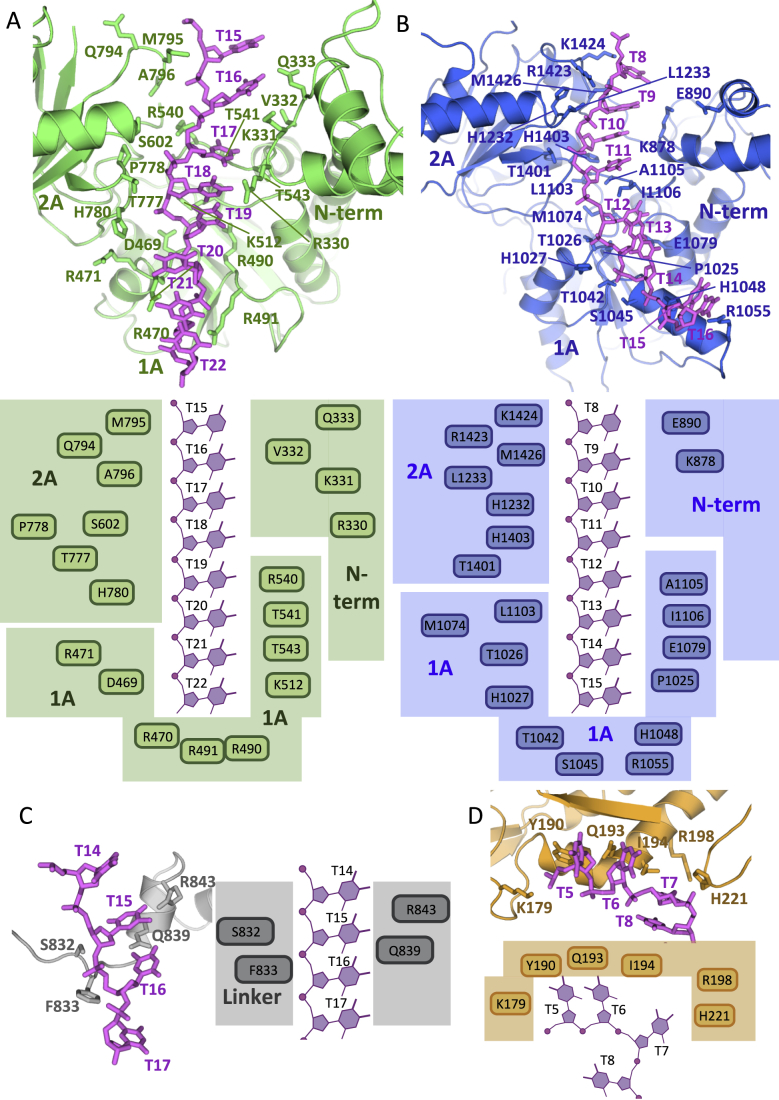
Details of Protein:ssDNA Interaction. Note that Interactions Involving the 2B/2B-like Sub-domains with ssDNA Are Described in the Main Text, Related to Figures 4, 6, and 7 (A) Interactions between the N-term, 1A and 2A sub-domains of the vestigial helicase domain and ssDNA. In the N-term sub-domain, residues Gln333, Val332, Lys331 and Arg330 interact with nucleotides T15, T16, T17 and T18. From the 1A sub-domain Thr541, Lys512, Thr543, Arg471, Asp469 and Arg540 contribute to binding of nucleotides T18, T19, T20, T21 and T22. In the 2A sub-domain, Thr777, Ala796, Met795, His780 and Pro778 interact with T15, T16, T17, T18 and T19. These nucleotides are also in close proximity to residues Gln794, and Ser602. Residues Arg470, Arg490, and Arg491 make hydrogen bond interactions with T21, T22. Upper and lower sub-panels provide 3D and schematic representations of the binding site, respectively. Stacking between bases is continuous between T15 and T19, where it is disrupted by W638. Stacking resumes between T20 and T22. (B) Interactions between the N-term, 1A and 2A sub-domains of the active helicase domain and ssDNA. Residues Glu890 and Lys878 in the N-term sub-domain make hydrogen bond interactions with nucleotides T8 and T9. From the 1A sub-domain, Pro1025, Thr1042, His1048, Arg1055, Glu1079, Ile1106, Ala1105, Leu1103 and Met1074 are in close proximity to nucleotides T11, T12, T13, T14 and T15. Residues Thr1026, His1027 and Ser1045 made hydrogen bond interactions with T14 and T15. From the 2A sub-domain, His1232, Leu1233, Lys1424, Arg1423 and Met1426 are in close proximity to nucleotides T8, T9, T10 and T11, with Thr1401 and His1403 also making hydrogen bond contacts with the same ssDNA region. Upper and lower sub-panels provide 3D and schematic representations of the binding site, respectively. Stacking between bases is observed between T8 and T11, is disrupted by insertion of A1105 and I1106, and resumes between T12 and T14. (C) Interactions between ssDNA and the linker residues between the vestigial and active helicase domains. Residues Ser832, Phe833, Gln839 and Arg843 make hydrophobic and polar contacts with nucleotides T15 and T16. Upper and lower sub-panels provide 3D and schematic representations of the binding site, respectively. (D) Interactions between the ssDNA and the trans-esterase domain. T8 emerges from the helicase tunnel where it is redirected by His221 (located in a loop between helices αF and αG) toward the ssDNA-binding site of the trans-esterase domain. T7 binds within the proximity of Arg198 and Ile194 while T6 and T5 interact with Lys179, Tyr190, and Gln193.

The ssDNA binds across the trans-esterase, vestigial, and active helicase domains (Figure 4A). Where it is bound to the helicase domains, it is almost entirely surrounded by the protein, an unprecedented observation in monomeric helicases (Figure 4B). In both SF1A and SF1B helicases, ssDNA has been observed bound across the top of the 1A and 2A domains, and this is indeed the case for ssDNA-binding at both the vestigial and active helicase domains (Figure 6A; also, see interaction details in Figure S5). Strikingly, the ssDNA at the 5′ end emerges from the helicase tunnel to be redirected at a 90° angle toward the trans-esterase domain by a loop between helices αF and αG in the trans-esterase domain (detailed in Figure S5D). At the very 5′ end, only two nucleotides are observed to interact with the trans-esterase domain (Figure S5D), but those interactions are essentially similar to those observed in the structure of the TraI trans-esterase domain alone bound to an ssDNA representative of the sequence 5′ to the *nic* site (Larkin et al., 2005).

In a previous study, sequence insertions were introduced in TraI, and their effects on conjugation were tested (Haft et al., 2006). Five of these mutants map to the ssDNA-binding interface of TraI as revealed by the structure presented here (see location in Figure S3C), and these have dramatic effects on conjugation, either abolishing it entirely or decreasing it substantially (Haft et al., 2006). In addition, we describe here another five mutants that we introduced in TraI by site-directed mutagenesis (see location in Figure S3D). These were tested for ssDNA-binding and conjugation and shown to affect either one (2- to 3-fold decrease) or the other (up to 60-fold difference) or both, thereby providing further validation of the structure (see details in Figure S3D).

**Figure 7:**
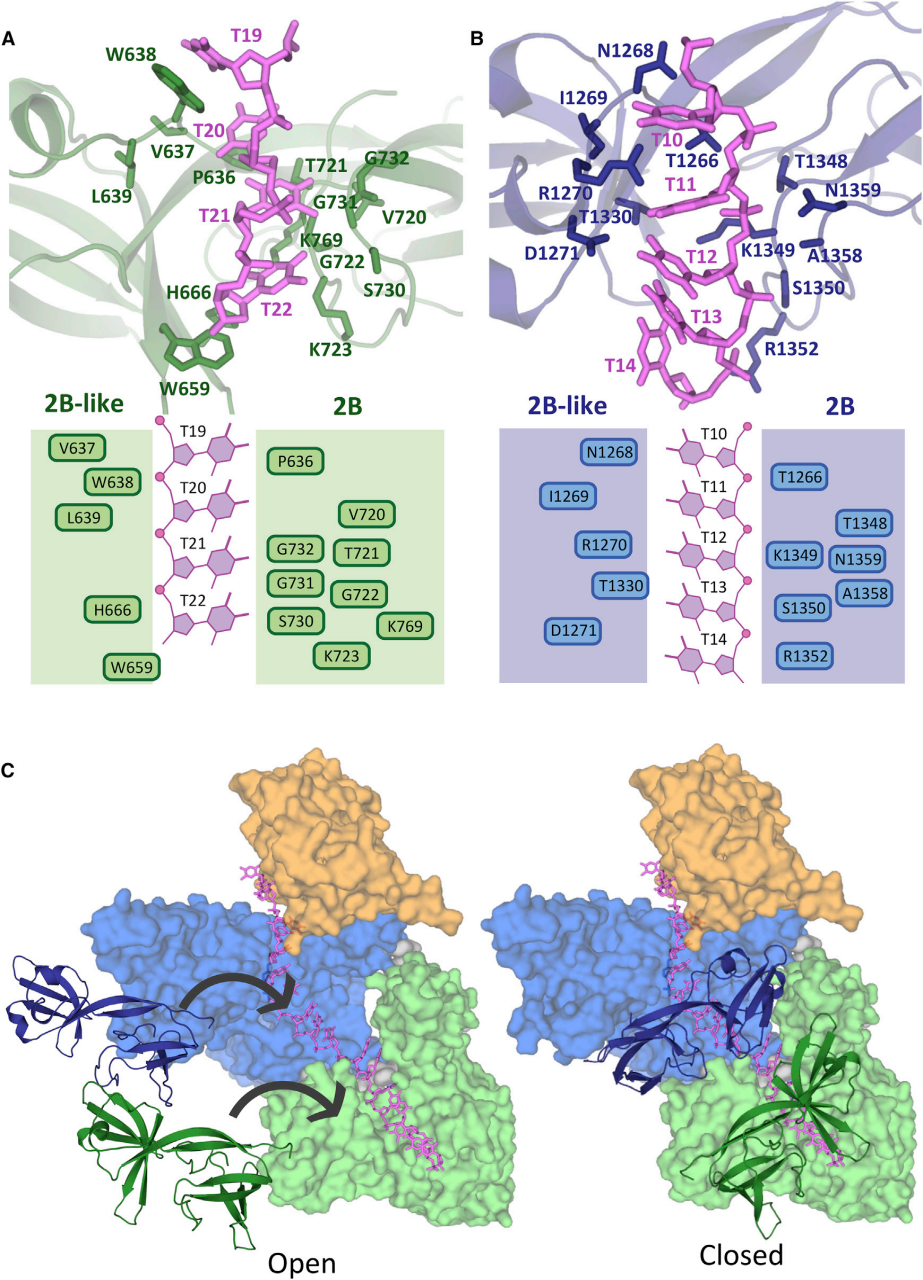
Role of 2B/2B-like Sub-domains in ssDNA-Binding (A) Details of ssDNA interaction with the 2B/2B-like sub-domain of the vestigial helicase domain. Top: Three-dimensional structure of the binding site with interacting residues and nucleotides labeled and shown in stick representation. Bottom: Schematic representation of the binding site. In brief, Trp638, Val637, Pro636, and Leu639 make hydrophobic contacts with nucleotides T19 and T20. His666, Trp659, Lys723, Ser730, Gly722, Lys769, Val720, and Thr721 make hydrophobic contacts with nucleotides T20, T21, and T22. Gly731 and Gly732 make hydrogen bond interactions with T21 via their main chain oxygen. (B) Details of ssDNA interaction with the 2B/2B-like sub-domain of the active helicase domain. Top and bottom are as in (A). Briefly, Thr1266, Ile1269, Arg1270, Thr1348, Asn1359, and Thr1330 makes contact with nucleotides T10 and T11. Also Ala1358, Lys1349, Ser1350, Arg1352, and Asp1271 are in close proximity to nucleotides T12, T13, and T14. Further Asn1268, Arg1352, and Asn1359 make hydrogen bond interaction with the ssDNA backbone. (C) Open and closed forms of the TraI 2B/2B-like sub-domains. Left: Hypothetical open form of TraI. Here, the 2B/2B-like sub-domains are shown in cartoon representation in dark green and dark blue as in Figure 6A. Arrows indicate closing motions to reach the closed state shown to the right. Right: Closed state of TraI represented by the structure of TraI bound to the 22-mer ssDNA. Color-coding of 2B/2B-like sub-domains is as in Figure 6A.

A unique feature of ssDNA-binding in TraI is the role played by the 2B/2B-like sub-domains in both helicase domains (Figure 7). Overall, the 2B/2B-like sub-domains together contribute 603 Å^2^ of surface area to binding of ssDNA; i.e., 32% of the total area buried upon ssDNA-binding. The ssDNA-binding site is located in a groove between the 2B and 2B-like parts of the sub-domains (Figures 7A and 7B). Strikingly, both these sub-domains appear to act as clamps holding the ssDNA in place (Figure 7C), resulting in the ssDNA being completely surrounded by protein. Indeed, each sub-domain is tethered to their respective 2A sub-domain by two short linker peptides that could act as hinges around which the entire 2B/2B-like sub-domain could pivot (as illustrated in Figure 7C). In the closed conformation observed in the structure, the ssDNA cannot access its binding platform, and thus, some opening of the structure is required. A hinge motion of the 2B/2B-like sub-domains appears most plausible and would indeed result in the binding site being exposed (Figure 7C, left). One could also hypothesize that the open form of TraI observed when either unbound, or bound to oligonucleotide sequences derived from sequences 5′ to the *nic* site (see above), might correspond to a structure where the 2B/2B-like sub-domains are swung out.

The clamping motion of the 2B/2B-like sub-domains on to the ssDNA might affect enzyme processivity. Indeed, the processivity of TraI is known to be very high (>850 base pairs), making it one of the most processive monomeric helicases known (Sikora et al., 2006). TraI also separates double-stranded DNA at a rate of 1,100 base pairs per second. This is consistent with the F or R1 plasmid needing to be unwound in a matter of minutes or entire bacterial genomes being transferred within 1.5 hr during conjugation (Sikora et al., 2006). It is conceivable that the concerted action of two clamping domains, the 2B/2B-like sub-domains, might result in a formidably active and processive enzyme.

In previous studies, the 2B/2B-like sub-domain of the TraI vestigial helicase domain has been identified as “translocation signal A” (TSA) since it contains sequences essential for the recruitment of TraI to the T4S system (Lang et al., 2010). It has been hypothesized to contain the protein-protein interaction surfaces mediating recruitment of TraI and the relaxosome to the T4S system. A second translocation signal was identified, TSB, and this sequence locates within the 2B/2B-like sub-domain of the active helicase domain (Lang et al., 2010). As explained above, both 2B/2B-like sub-domains also interact with ssDNA and act as clamps that close in on the ssDNA. Thus, the 2B/2B-like sub-domains play two major roles in relaxase function: (1) interacting with the DNA and possibly promoting high processivity and (2) mediating recruitment of the relaxosome to the T4S system. As would be expected, the surfaces identified here to interact with ssDNA and those identified previously to be involved in recruitment to the T4S system are on different sides of the 2B/2B-like sub-domain structures (Figure S4E).

The binding mode of ssDNA on TraI also explains why binding of TraI to sequences 5′ or 3′ to the *nic* site of *oriT* is mutually exclusive. Indeed, negative cooperativity between binding at the trans-esterase binding site (where the sequence 5′ to the *nic* site binds) and binding at the helicase domains (where the sequence 3′ to the *nic* site binds) has been observed, but its structural basis was unknown (Dostál and Schildbach, 2010). Also, the biochemical experiments described here clearly demonstrate that binding to one or the other site results in TraI adopting either a closed or open conformation and that two TraI molecules occupy *oriT* on either side of the *nic* site. Although there is no report of the structure of a full-length relaxase with ssDNA bound to its trans-esterase domain, the structures of the trans-esterase domain of both TraI (Larkin et al., 2005) and another relaxase, TrwC (Guasch et al., 2003), have been determined. That of TrwC is particularly instructive because it contains the ssDNA sequence 5′ of *nic* up to the *nic* cleavage site. The TrwC trans-esterase domain superimposes very well on the trans-esterase domain of TraI (Figure S4B), and, therefore, the ssDNA sequence bound to TrwC can be easily mapped onto the full-length TraI structure presented here (Figure 6B). As can be seen from Figure 6B, this DNA would clash with the part of the 22-mer ssDNA that we observe bound to the trans-esterase domain in our TraI:ssDNA complex structure (as shown in Figure 6B, right). Thus, the structure presented here clarifies the structural basis of negative cooperativity.

**Figure S6:**
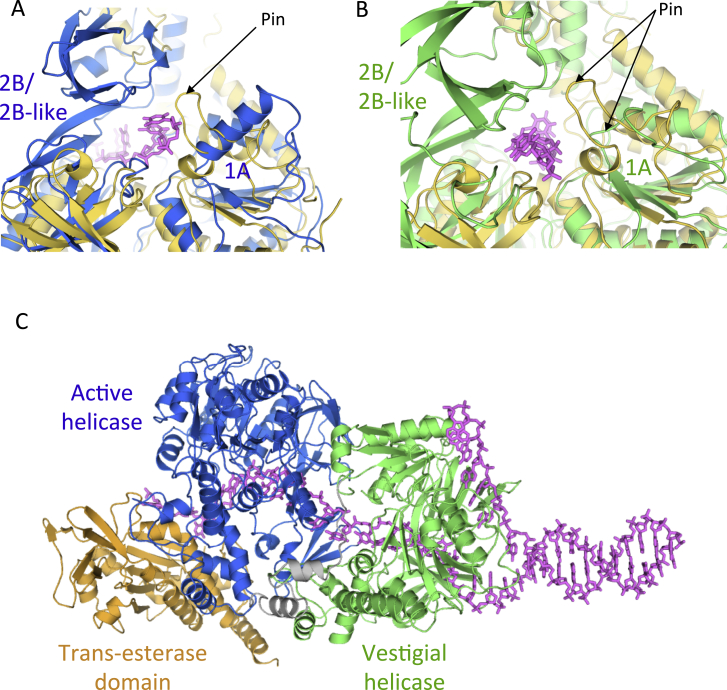
Potential Location for the Pin Loop of TraI, Related to Figure 4 (A) Superposition of the TraI active helicase domain with RecD2, in a view that is 180° away from the view shown in Figure S4C, right panel. Color-coding and representation are the same as in Figure S4C, right panel. The pin loop of RecD2 is indicated. In this TraI domain, this loop is not present. (B) Superposition of the TraI vestigial helicase domain with RecD2, in view that is 180° away from the view shown in Figure S4C, left panel. Color-coding and representation are the same as in Figure S4C, left panel. The pin loop of RecD2 is indicated. An equivalent loop is present in this TraI domain and might act as a potential pin loop in TraI. (C) Model of TraI bound to a ds/ssDNA duplex. This ribbon and stick representation of TraI splitting a duplex DNA was generated using COOT (Emsley et al., 2010) and CHIMERA (Pettersen et al., 2004). It is meant to illustrate where strand splitting occurs during TraI-mediated unwinding.

Monomeric helicases usually contain a so-called pin loop, which serves to splay apart the two strands of the double-stranded DNA (dsDNA). In RecD2, this pin loop locates to a structure in its 1A domain (Figures S6A and S6B). In the active helicase domain of TraI, this structure is lacking (Figure S6A), but a similarly positioned loop is observed in its vestigial helicase domain between β3 and αH (Figure S6B). Whether this loop acts as a pin loop remains to be experimentally determined. Nevertheless, similarity in pin loop positioning may suggest that the splitting of the dsDNA occurs at the vestigial helicase domain. Thus, TraI is an ssDNA translocase that uses its active helicase domain to thread ssDNA through while splitting the dsDNA at the vestigial helicase domain (Figure S6C).

## Conclusion

The structure presented here provides unique insights into the mechanism of relaxases, proteins essential to the conjugation process that drives adaptation and evolution in bacteria, and of immense biomedical importance since it is the main driver for the spread of antibiotic resistance genes.

Relaxases have been the subject of intensive biochemical and structural studies, but most have been based on identifying the properties and structures of domains alone. Those using the full-length relaxases were aimed at characterizing the binding and reaction parameters of the various activities, not the structures. More recently, it has been suggested that two relaxases might be needed to carry out their function (Dostál et al., 2011); however, a dimer has never been experimentally observed, and the mechanism by which two relaxases could come together at *oriT* has remained unclear. The biochemical and structural characterization of TraI reported here demonstrates that two molecules of TraI can bind to the *oriT* DNA and that this DNA-mediated dimer is stable as it can be purified to homogeneity. It shows that, in this dimer, the two TraI molecules bind on each side of the *nic* site, the molecule binding on the 3′ side of *nic* adopting a closed, “helicase,” conformation, while that binding on the 5′ side adopts an open, “trans-esterase,” conformation. The structure explains why one molecule of TraI cannot bind to both sites simultaneously and, therefore, that two TraI molecules might be needed to operate on *oriT*. The reason why TraI should support two activities, trans-esterase and helicase, on the same polypeptide could be that the TraI molecule transferred to the recipient cell may switch to a ssDNA translocase mode in order to pull the ssDNA into the recipient cell and complete plasmid transfer more efficiently.

**Figure S7:**
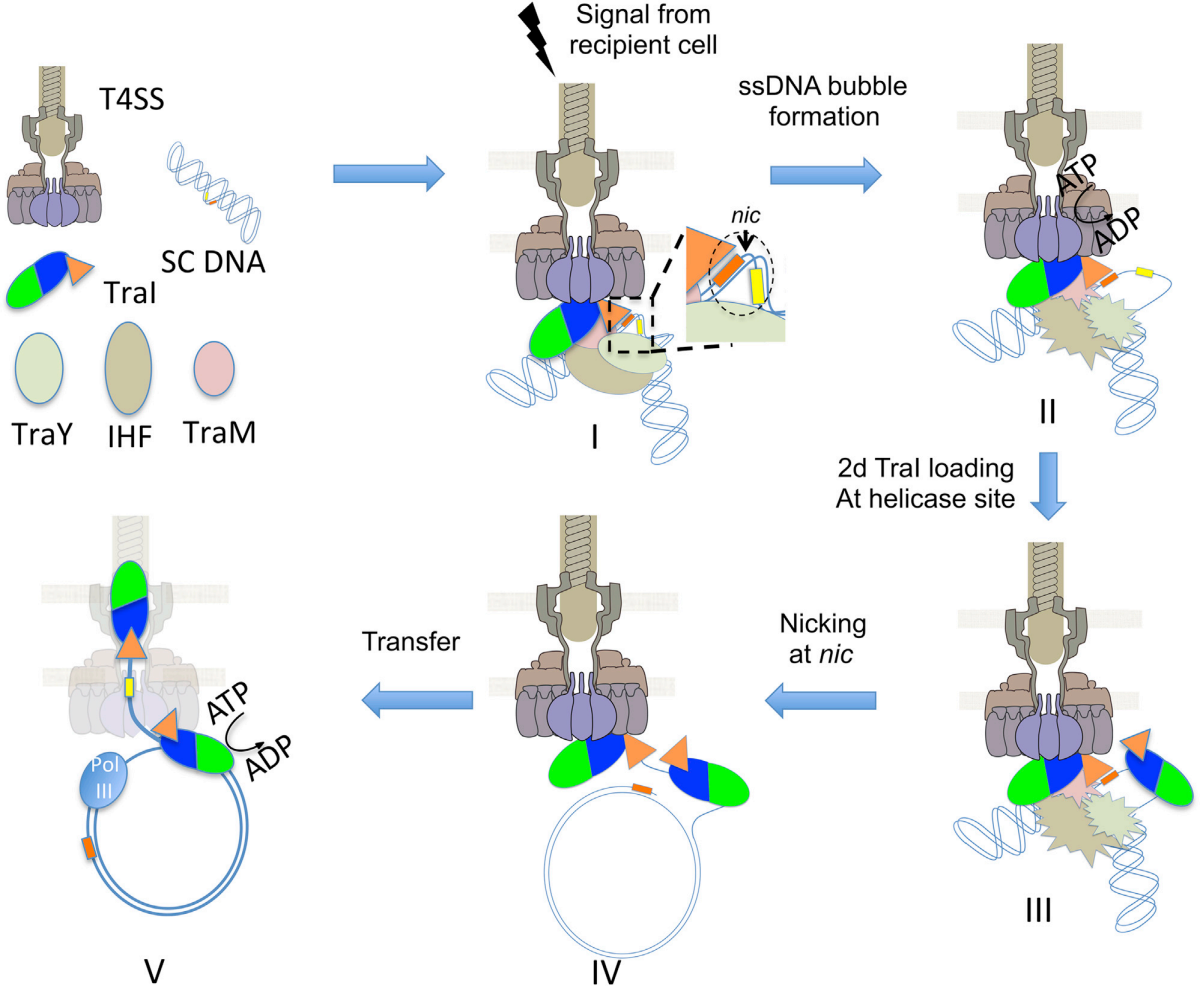
Related to Figures 2 and 4 Schematic diagram of events taking place in the donor cell during conjugation. The pre-initiation complex (state I) is formed from components shown at left. The details of the *oriT* region are shown in the zoom-in inset at right of the pre-initiation complex. In this zoom-in inset as in all panels and states of Figure S7, the red and yellow rectangles represent the sequence 5′ and 3′ of *nic*, respectively. In the zoom-in inset, the dashed circle locates *oriT* and the position of the *nic* site is indicated by the arrow. The states II-V and steps leading to their formation are described and discussed in the main text. The conformational change in the relaxosome proteins in state II are indicated by spiked ovals. In the F/R1 family plasmid systems, the relaxosome is composed of the plasmid-encoded relaxase TraI, the plasmid-encoded accessory proteins TraM and TraY, and the chromosome-encoded accessory protein IHF.

Thus, mechanistic insight informing on the sequence of events taking place in the donor cell during conjugation emerges from the results presented here and is recapitulated in the scheme presented in Figure S7. All evidence published so far has convincingly shown that a pre-initiation complex consisting of the relaxosome docked onto a T4S system is formed constitutively before conjugative transfer starts (state I in Figure S7) (Lang et al., 2011, Lang and Zechner, 2012, Mihajlovic et al., 2009, Sut et al., 2009). In the pre-initiation complex, one molecule of TraI is bound to super-coiled *oriT* 5′ of *nic*, which is a poor substrate for the nicking activity. At this stage, the pre-initiation complex lays dormant. Upon mating—contact with a recipient cell—a signal would lead to activation of the T4S system and the relaxosome, resulting in the formation of an ssDNA bubble around the *nic* site (state II in Figure S7), perhaps through the activation of T4S system ATPases. With the sequence 3′ of *nic* now single stranded, a second TraI molecule can bind, in its helicase mode (state III in Figure S7), and unwinding starts. Concomitantly, the previously bound TraI molecule catalyzes the nicking reaction, covalently attaches to the resulting free 5′ phosphate, and transfers through the T4S system, while the resulting free 3′ OH engages with the PolIII machinery (states IV and V in Figure S7).

TraI unwinding capability is truly remarkable: it exhibits high processivity but also a large step size compared to other helicases (Sikora et al., 2006). Processivity is likely due to the unique property of the TraI helicase domains to wrap around the ssDNA. The large step size might be related to the large footprint of ssDNA binding onto the relaxase, one of the largest in all monomeric helicases, extending to over 14 nucleotides. Remarkably, the rate at which TraI is capable of translocating along an ssDNA correlates well with the rate at which ssDNA transfers to the recipient cell. It has been suggested (but never demonstrated) that T4S system ATPases mediate ssDNA transfer through the system by acting as T4S system-tethered translocases for the ssDNA, a prime candidate for this role being VirD4, one of the three T4S system ATPases (Ilangovan et al., 2015). If this is correct, then the rate at which TraI and VirD4 operate must be closely coupled.

Relaxases are one of the most conserved components of conjugative systems. Because they drive the spread of antibiotic resistance genes among bacterial populations (not only among pathogens but also within commensal microbes), relaxases are prime targets for inhibition. However, the lack of structural information on how its various domains cooperate in function has been a major obstacle in deriving mechanistic insights that could be exploited for inhibitor design. With the structure reported here, renewed efforts in designing means to inhibit horizontal gene transfer can now proceed.

## STAR★Methods

### Key Resources Table

**Table:** 

REAGENT or RESOURCE	SOURCE	IDENTIFIER
**Bacterial and Virus Strains**		

*Escherichia coli* DH5α	ThermoFisher Scientific	N/A
*Escherichia coli* MS411	M. Schembri, DTU, Denmark	N/A
*Escherichia coli* MS614	M. Schembri, DTU, Denmark	N/A
One Shot BL21 Star (DE3)	ThermoFisher Scientific	N/A
One Shot TOP10	ThermoFisher Scientific	N/A

**Chemicals, Peptides, and Recombinant Proteins**		

Trypsin	Sigma	T1426; CAS: 9002-07-7
3-(2-Iodoacetamido)-PROXYL	Sigma	253421; CAS: 27048-01-7
D8-glycerol	Sigma	G5516; CAS: 56-81-5
Common lab reagents	N/A	N/A

**Deposited Data**		

TraI relaxase cryo EM map	This study	EMD-3601
TraI relaxase cryo EM structure	This study	5N8O

**Oligonucleotides**		

49-mer -GCAAAAACTTGTTTTTGCGTGGGGTGTGGTGCTTTTGGTGGTGAGAACC	This study	Eurofins Genomics
30-mer - GCAAAAACTTGTTTTTGCGTGGGGTGTGGT	This study	Eurofins Genomics
22-mer - GGTGCTTTTGGTGGTGAGAACC	This study	Eurofins Genomics
19-mer - GCTTTTGGTGGTGAGAACC	This study	Eurofins Genomics
49-mer_Cy5∗6-FAM_ – Cy5-GCAAAAACTTGTTTTTGCGTGGGGTGTGGT-PClinkerC3- GCTTTTGGTGGTGAGAACC-6-FAM	This study	Genelink
22-mer_Alexa488_ – GGTGCTTTTGGTGGTGAGAACC-Alexa488	This study	Eurofins Genomics
22-mer_5′SH_ – SH-GGTGCTTTTGGTGGTGAGAACC	This study	Genelink
22-mer_3′SH_ - GGTGCTTTTGGTGGTGAGAACC-SH	This study	Genelink
Primers for TraI single- and double- mutant in pCDF and pHP2 plasmid, see Table S1	This study	N/A

**Recombinant DNA**		

pCDF::TraI	This paper	N/A
pCDF::TraI_1475_	This paper	N/A
pCDF::TraI-MBP	This paper	N/A
R1-16Δ*traI*	Lang et al., 2010	N/A
pHP2	Zechner et al., 1997	N/A

**Software and Algorithms**		

MOTIONCORR 2.1	Li et al., 2013	http://cryoem.ucsf.edu/software/driftcorr.html
CTFFIND4	Rohou and Grigorieff, 2015	http://grigoriefflab.janelia.org/ctffind4
E2BOXER	Tang et al., 2007	http://blake.bcm.edu/emanwiki/EMAN2/Programs/e2boxer
RELION 1.4	Scheres, 2012	http://www2.mrc-lmb.cam.ac.uk/relion/index.php/Main_Page
MOTIONCOR2	Unpublished	http://msg.ucsf.edu/em/software/motioncor2.html
GAUTOMATCH	Unpublished	http://www.mrc-lmb.cam.ac.uk/kzhang/
RELION 2.0	Scheres Lab	http://www2.mrc-lmb.cam.ac.uk/relion/index.php/Main_Page
Chimera	Pettersen et al., 2004	https://www.cgl.ucsf.edu/chimera/
MODELER	Webb and Sali, 2016	https://salilab.org/modeller/
COOT	Emsley et al., 2010	https://www2.mrc-lmb.cam.ac.uk/personal/pemsley/coot/
PHENIX	Adams et al., 2010	https://www.phenix-online.org
Molprobity	Chen et al., 2010	https://www.phenix-online.org
DeerAnalysis2015	Jeschke et al., 2006	http://www.epr.ethz.ch/software.html

### Contact for Reagent and Resource Sharing

Further information and requests for resources and reagents should be directed to and will be fulfilled by the Lead Contact, Gabriel Waksman (g.waksman@mail.cryst.bbk.ac.uk).

### Method Details

#### Cloning, expression and purification of TraI and TraI variants (TraI-MBP, and TraI_1-1475_), and TraI single site mutants

All TraI constructs described in this study (TraI; TraI-MBP and TraI_1-1475_) were cloned into a pCDF1b expression vector (Novagen) where protein expression is controlled by a *lac* promoter. All constructs include an N-terminal 6 Histidine tag (His_6_-tag) along with an enterokinase cleavage site between the tag and TraI. For the TraI construct, the full-length sequence was amplified by PCR from a TraI clone, pHP2, described in (Zechner et al., 1997). The resulting full-length *traI* pCDF clone was used as template to generate the C-terminal deletion construct TraI_1-1475_. For TraI-MBP, the sequence encoding MBP together with that encoding a small 5 amino acid linker (amino acid sequence ‘TPGSP’) was added at the 3′ end of the full-length *traI* construct. All TraI constructs and single- or double- site mutation TraI variants were generated using the In-fusion cloning method (Clontech).

The expression and purification of all TraI and TraI variant proteins described here were performed as described below. Freshly transformed *E.coli* BL21^∗^ cells containing the pCDF1b::TraI and TraI variants were grown in 1 L of LB media to an OD_600_ of 0.7, expression induced using 1mM IPTG (isopropyl β-D-1-thiogalactopyranoside) and incubated for 16 hr at 17°C. The cells were harvested by centrifugation and resuspended in a buffer containing 50 mM Tris (pH 7.2), 100 mM NaCl, and a complete mini EDTA-free protease inhibitor cocktail tablet (Roche) followed by lysis through an EmulsiFlex-C5 homogenizer. The cell lysate was centrifuged at 17000 g for 30 min and the resulting supernatant loaded onto a nickel affinity column His trap (GE Healthcare). All TraI and TraI variants were eluted from the column using an imidazole gradient (50 mM Tris (pH 7.2), 100 mM NaCl, 1 M Imidazole). The eluted protein was dialysed against 50 mM Tris (pH 7.2), 50 mM NaCl for 4 hr. The dialysed protein sample was loaded onto an anion exchange Hi Trap Q column (GE Healthcare) and eluted using a 50mM-1M NaCl gradient in 50 mM Tris (pH 7.2). The fractions containing the protein of interest were then concentrated and subjected to size exclusion chromatography using a Superdex 200 10/300 column (GE Healthcare) in a buffer containing 50 mM Tris (pH 7.2) and 100 mM NaCl as the mobile phase. The proteins purified after size exclusion chromatography were flash frozen stored at −80°C.

#### Formation and purification of TraI and TraI variant bound to the 19-mer, 22-mer, and 30-mer ssDNA

The ssDNA oligonucleotides used in this study were purchased commercially (Eurofins Genomics) and stocks of these were prepared at 100 μM concentration using 50 mM Tris (pH 7.2), 100 mM NaCl buffer and stored at −20°C. The purified protein and ssDNA oligos were mixed in 1:1.5 (protein:ssDNA) molar ratio and incubated on ice for 10 min. The TraI:ssDNA (1:1) complex was then purified by size exclusion chromatography using a Superdex 200 10/300 column to remove any unbound ssDNA.

#### Formation and purification of the various TraI and TraI variants dimers bound to the 49-mer ssDNA

For the dimer titration experiment described in Figure 2A, different protein:ssDNA molar ratios (1:1, 2:1, 4:1) were mixed to a final protein concentration of 1 mg/mL and a final volume of 100 μL. The samples were loaded consecutively onto a Superdex 200 10/300 size exclusion chromatography column to assess ssDNA-mediated oligomerization of TraI.

#### Mild proteolysis studies of TraI:ssDNA complexes

Purified TraI was mixed with various ssDNA oligonucleotides (19-mer, 22-mer, 30-mer and 49-mer) in a 1:1.2 (protein:ssDNA) molar ratio to a final protein concentration of 1 mg/mL followed by incubation on ice for 10 min. All samples were mixed with bovine trypsin (Sigma) in a 100:1 TraI:trypsin (w/w) ratio and incubated at 20°C. Aliquots were taken at 30, 60 and 120 min, mixed with 4 times concentrated SDS-PAGE loading buffer (in a ratio of 3:1 volume per volume of protein:buffer), and heated at 95°C for 5 min to stop the proteolysis reaction. The collected samples along with a non-proteolysed TraI were run on an SDS-PAGE.

#### Design and analysis of the photocleavable TraI:ssDNA complexes

The modified oligonucleotide 49-mer_Cy5∗6-FAM_ was purchased commercially (Genelink). Its design is described in the main text. The photocleavable site is commercially known as PC linker C3. PC Linker C3 (chemical name: 3-(4,4’-Dimethoxytrityl)-1-(2-nitrophenyl)-propan-1-yl-[(2-cyanoethyl)-(N,N-diisopropyl)]-phosphoramidite) contains a non-nucleosidic moiety that can be used to link two nucleotide sequences through a short, UV photo-cleavable C3 spacer arm. Purified TraI-MBP protein and 49-mer_Cy5∗6-FAM_ were mixed in a 1:1.5 protein:ssDNA molar ratio to a final protein concentration of 5 mg/mL, followed by incubation on ice for 10 min. The TraI-MBP:49-mer_Cy5∗6-FAM_ 1:1 (protein:ssDNA) complex was purified using a Superdex 200 10/300 gel filtration column removing any excess of unbound 49-mer_Cy5∗6-FAM_. The purified TraI-MBP:49-mer_Cy5∗6-FAM_ was next concentrated and mixed with a 3 to 1 molar excess of pure TraI_1-1475_ and incubated on ice for 10 min. The TraI-MBP:49-mer_Cy5∗6-FAM_:TraI_1-1475_ complex was then purified by gel filtration, thereby removing the excess of unbound TraI_1-1475_. The purified TraI-MBP:49-mer_Cy5∗6-FAM_:TraI_1-1475_ complex was then subjected to photocleavage at UV 365 nm on ice using 3 alternating on/off UV cycles (5 min on, 5 min off per cycle). The photocleaved sample was then concentrated and analyzed by gel filtration using a Superdex 200 10/300 column. Absorbance at 280 nm for protein, 649 nm for Cy5 and 497 nm for 6-FAM was monitored throughout.

#### ssDNA binding assay

A modified 22-mer with a 3′ Alexa488 label (22-mer_Alexa488_) was purchased (Eurofins Genomics) and fluorescence anisotropy of the 22-mer_Alexa488_ at final concentration of 10 nM was measured in the presence of increasing concentration of TraI wild-type or TraI mutant proteins (TraI single site mutants Y190A, R330A, M795A, A1105W and double mutant A1105W/M795A). Data were recorded using a Synergy 2 Multi-mode Reader (BioTek). Excitation wavelength was 485 nm (slit width of 20 nm) and emission wavelength was 540 nm (slit width of 25 nm). The 22-mer_Alexa488_ and TraI wild-type/mutants were mixed in 50 mM Tris (pH 7.2), 100 mM NaCl buffer and incubated at 25°C for 20 min prior measurement. Binding assay of each wild-type and mutants were carried out in triplicates. The data was corrected for background Alexa488 emissions. The normalized data was fit to a “Saturation binding - single site non specific binding” curve using Graphpad Prism 6.0.

#### Conjugation assays

Conjugation assays were performed as described in (Lang et al., 2014) with mutations introduced into pHP2. Conjugation frequencies were calculated as transconjugant cells per donor cells from at least 3 independent experiments. Normal levels of protein production from the mutant *traI* alleles relative to wild-type was confirmed by immunochemical detection and western blotting, as described previously.

#### Cryo-EM sample preparation and data collection

Samples of 4 μL of purified TraI/22-mer ssDNA complex at a concentration of 1mg/mL, with or without 1 mM AMP-PNP/Mg^2+^, were deposited onto glow-discharged lacey carbon grids or Quantifoil R1.2/1.3 gold grids, and plunge-frozen using a Vitrobot, with blot force set to 1, blot time of 4 s, 100% humidity and at room temperature. Grids were imaged in two different conditions: in house using a FEI Tecnai G2 Polara transmission electron microscope and at the eBIC facility at the Diamond synchrotron using a Titan Krios. Both microscopes were operated at 300 kV, and in both cases data was collected on a K2 Summit direct electron detector operated in counting mode, and placed at the end of a Quantum energy filter operated with a slit width of 20 eV. Data on the Polara was acquired manually using SerialEM. It had a final pixel size of 1.86 Å, and was collected using a dose of ∼7 e^-^/(pix^∗^sec) (equivalent to ∼2 e^-^/Å^2^^∗^sec at the specimen level) and a total exposure of 19 s (∼40 e-/Å^2^) divided into 76 frames. Data on the Krios was automatically acquired with EPU software (FEI). It had a final pixel size of 1.05 Å, and was collected using a dose of 6.3 e^-^/(pix^∗^sec) (equivalent to 5.7 e^-^/Å^2^^∗^sec at the specimen level) and a total exposure of 8 s (∼45 e-/Å^2^) divided into 20 frames. In both cases defocus ranged between 2 and 3.5 μm.

#### Cryo-EM image processing and reconstruction

In house Polara data was first used to generate a ∼7 Å resolution map. Krios data was then collected on the TraI:ssDNA complex without AMP-PNP and used to generate a ∼4.5 Å resolution map. Finally, a second Krios dataset was collected on the TraI:ssDNA complex containing AMP-PNP in the vitrification buffer and used to generate a 3.9 Å resolution map.

Polara data: A total of 820 micrograph movies were aligned using MOTIONCORR 2.1 (Li et al., 2013) and CTF was estimated using CTFFIND4 (Rohou and Grigorieff, 2015). An initial dataset of ∼118,000 particles was semi-automatically picked from 350 micrographs using E2BOXER (Tang et al., 2007) and subjected to 2D classification in RELION 1.4 (Scheres, 2012). The best classes (from 26,500 particles) showed high-resolution features and were used to generate 10 starting models using the “initial model” feature of the EMAN2 project manager. All of the starting models obtained were used to refine the same 26,500 particle subset in RELION 1.4, using an initial low-pass filter of 60 Å. Two of the models led the data to converge to similar structures, each to a resolution of ∼10 Å. A bigger dataset of ∼220,000 particles was picked from the entire micrograph dataset with E2BOXER and subjected to rounds of 2D and 3D classification, from which the best 32,700 particles were selected and refined against one of the 10 Å models low-pass filtered to 40 Å. This exercise yielded a structure with a resolution of 7 Å, where we could clearly distinguish rod-shaped densities for α helices, and flat densities occupied by β sheets.

Krios dataset 1: A total of 4035 micrograph movies were aligned using MOTIONCORR 2.1, and CTF was estimated using CTFFIND4. Thon rings could be visible up to resolutions ranging between 7 and 3 Å, with 16% of micrographs displaying thon rings beyond 4 Å, 34% between 4 and 5 Å, 30% between 5 and 6 Å, and 20% above 6 Å. Circa 950,000 particles were semi-automatically picked using E2BOXER, divided in 3 pools for best use of computational resources and subjected to 2D and 3D classification in RELION 1.4 to select the best subsets. The previously-determined 7 Å map was used as starting reference, filtered to 20 Å. A selected combined dataset of 328,000 particles was subjected to 3D refinement. Alignments were further improved by rebalancing particle orientations (eliminating particles from preferential view that has worse CTFFIND4 scores), and by refining particles within a mask calculated with threshold level 0.008, 5 pixel edge extension and 5 pixel soft drop off to eliminate noise around the reference. The map was post-processed in RELION to sharpen high resolution features, using a mask calculated with threshold level 0.01, 3 pixel edge extension and 3 pixel soft drop off. The final resolution for this map was 4.5 Å as judged from the mask corrected FSC using the 0.143 threshold criterion (not shown).

Krios dataset 2: A total of 2916 micrograph movies were aligned using MOTIONCOR2 (developed by Zheng, Q., Palovcak, E., Armache, J-P., Cheng, Y., and Agard, D.A., UCSF, San Francisco, USA), and CTF was estimated using CTFFIND4. Thon rings could be visible up to resolutions ranging between 7 and 3 Å, but the overall quality of the data was significantly better than the previous data collection: 57.5% of micrographs displayed thon rings beyond 4 Å, 23.5% between 4 and 5 Å, 11% between 5 and 6 Å, and 8% above 6 Å. Circa 830,000 particles were automatically picked using GAUTOMATCH (developed by Zhang, K., MRC Laboratory of Molecular Biology, Cambridge, UK), and subjected to 3D classification in RELION 2.0 to select the best subsets. The previously-determined 4.5 Å map was used as starting reference, filtered to 40 Å. A selected combined dataset of approximately 184400 particles was subjected to 3D refinement in RELION 2.0, yielding a map at 4.0 Å resolution as judged from the FSC between independently refined unmasked and unfiltered half maps, using the 0.143 criterion, and which did not show any extra density for AMP-PNP. The other 3D classes did not refine to high resolution. Selected particles were re-extracted from micrographs corrected with MOTIONCOR2 with dose-weighting applied, and refined within a circular mask of 200 Å diameter. Post-processing in RELION using a mask with threshold 0.005, edge extension and dropoff of 3 pixels, and low-pass filter at 15 Å yielded a map at 3.9 Å resolution as judged from the mask-corrected FSC using the 0.143 criterion (Figure S1D). Local resolution analysis indicated that the vast majority of map voxels had a resolution around 3.5 Å, going as high as 3.1 Å in some regions (Figure S1C). The accession number for the 3.9 Å structure reported in this paper is EMDB: EMD-3601.

#### Model building and refinement

The electron density was clearly interpretable and a structural model could have been built *de novo.* However, when available, the use of homology models increases greatly the speed at which structural models can be built and fitted within an electron density. Thus, an initial model for TraI was first generated by rigid body fitting pre-existing crystal and NMR structures as well as structural homology models of various sub-domains into the 4.5 Å resolution EM map (Pettersen et al., 2004). For the trans-esterase domain the F/R1 crystal structure (2A0I) (Larkin et al., 2005) was used. For the vestigial helicase domain, we used the crystal structure of TSA containing sub-domains 2B/2B-like and part of 2A (4L0J) (Redzej et al., 2013) as well as the NMR structure of sub-domain 1A (2L8B) (Wright et al., 2012). For the active helicase domain, however, no experimentally derived structures were available. Therefore, for this domain, homology models of individual sub-domains (1A, 2A and 2B/2B-like) were generated using MODELLER (Webb and Sali, 2016), based on the structures of the RecD2 1A and 2A sub-domains (Saikrishnan et al., 2009) and on the structure of the TraI TSA sub-domain. All models were then rebuilt from the start and residues fitted into the electron density. The density was also clear enough to trace the protein chain in the regions linking the various sub-domains and also in the regions linking the trans-esterase and vestigial helicase domains and the vestigial and active helicase domains, and thus a model was built from residue 1 to 1473, leaving out the 283 residues of the C-terminal domain, and also leaving out a few regions where the electron density was either unclear or absent. These regions correspond to residues 236-267 and 307-314.

A clear and continuous density corresponding to ssDNA could be readily observed through the center of the structure, spanning both the vestigial and active helicase domains and in which the ssDNA was manually built using COOT. The density was clear enough to fit 18 nucleotides numbered 5 to 22. A poly thymine (poly-T) ssDNA was built as the purine and pyrimidine rings were not distinguishable enough to assign the correct 22-mer sequence.

The protein:ssDNA model was then subjected to iterative rounds of manual adjustment and rebuilding into the electron density using COOT (Emsley et al., 2010), stereo-chemical corrections and real space refinement using PHENIX (Adams et al., 2010). Progress in refinement was tracked through Ramachandran plots and Molprobity (Chen et al., 2010). The accession number for the coordinates of the model reported in this paper is PDB: 5N8O.

#### EPR Spectroscopy

The mutant TraI_R752C_ was grown expressed and purified as described above except that 2.5 mM dithiothreitol (DTT) was used in all buffers. Two 22-mer ssDNA thiolated at the 5′ end (22-mer_5′SH_) or at the 3′ end (22-mer_3′SH_) were commercially purchased (Genelink) and their 1:1 complexes with TraI_R752C_ were formed and purified by gel filtration in a buffer containing PBS pH 7.4 and 1 mM MgCl_2_. The protein:ssDNA complexes were adjusted to a concentration of 20 μM. Twenty times equivalent of spin label 3-(2-Iodoacetamido)-PROXYL (Sigma) was added in a final volume of 500 μL. The mixtures of protein:ssDNA complex and spin label were incubated overnight at 4°C and excess label was then removed by gel filtration using a Superdex200 10/300 column (GE healthcare). The samples were concentrated to a volume 70-80 μL and exchanged into D_2_O buffer using Amicon Ultra – 0.5 mL (50 kDa cutoff) centrifugal filters (Merck) and finally supplemented with deuterated D8-glycerol to a final concentration of 20% (v/v).

Continuous-wave EPR spectra were recorded with an E-Scan desktop spectrometer (Bruker) operating at 9.76 GHz and at room temperature. All measurements were carried out with 1 mW microwave power, 86 kHz modulation frequency, 0.1 mT modulation amplitude and 40 ms conversion time and 20 ms time constant.

EPR-based distance measurements were performed at 34 GHz (Q-Band) with an ELEXSYS E580 spectrometer equipped with an ER 5106QT-2w resonator (Bruker). A closed cycle cryostat (Cryogenic Limited) was used to maintain the temperature at 50 K. The 4-pulse DEER sequence (Pannier et al., 2000) used was π/2(ν_*obs*_)-τ_l_-π(ν_*obs*_)-t-π(ν_*pump*_)-(τ_l_+τ_2_-t)- π(ν_*obs*_)-τ_2_-*echo*, where the observer pulse length was 16 ns for π/2 and 32 ns for π pulses, τ_l_ = 200 ns, τ_2_ = 4500 ns and ν_*pump*_ = 22 ns.

The pump pulse frequency was set at the point of maximum signal in the field-swept echo spectrum and the frequency of the observer pulses was offset by −80 MHz. Two-step phase cycling was used to eliminate receiver offsets. The dipolar spectra were analyzed using the program DeerAnalysis2015 (Jeschke et al., 2006) and the distance distribution evaluated by Tikhonov regularization.

### Quantification and Statistical Analysis

Quantification and statistical analyses employed in this publication pertain to the analysis on electron microscopy data and the determination of structures by electron microscopy, which are integral parts of existing algorithms and software used.

### Data and Software Availability

The cryo-EM data and the structure coordinate were deposited in the EMDB and PDB with entry codes EMDB: EMD-3601 and PDB: 5N8O.

## Author Contributions

A.I. purified TraI and TraI variants, executed all biochemical experiments described, and built and refined the TraI:ssDNA model. S.R. and E.L.Z. planned and performed mutational and functional analysis of TraI in conjugation. G.Z. prepared cryo-EM grid, collected EM data, and performed the image analysis and carried out the EM reconstructions. C.W.M.K., E.S., and H.E.M. conducted EPR experiments and analyzed the data. G.W. supervised the work, made figures, and wrote the manuscript with contributions from E.L.Z., C.W.M.K., A.I., and G.Z.

## Supplemental Information

Table S1. Oligonucleotides Used in this Study for the Construction of Mutants, Related to Figure S3
