# Supplementary material for: Cryo-EM Structure of a Relaxase Reveals the Molecular Basis of DNA Unwinding during Bacterial Conjugation
Source: Cell. 2017 May 4;169(4):708–721.e12. doi: 10.1016/j.cell.2017.04.010 (PMC5422253; doi:10.1016/j.cell.2017.04.010)
Supplement: Table S1. Oligonucleotides Used in this Study for the Construction of Mutants, Related to Figure S3 [file mmc1.pdf]

Cell, Volume *169*

Supplemental Information

Cryo-EM Structure of a Relaxase Reveals the Molecular Basis of DNA Unwinding during Bacterial Conjugation

Aravindan Ilangovan, Christopher W.M. Kay, Sandro Roier, Hassane El Mkami, Enrico Salvadori, Ellen L. Zechner, Giulia Zanetti, and Gabriel Waksman

Table S1 – Oligonucleotides used in this study for the construction of mutants. Related to Figure S3.

Purpose	Site of Mutation	Sequence
TraI single and double site mutants in pCDF for ssDNA binding assay	Y190A	For – AATGTGGCCGCTAATCAGATTGCC<br>Rev – ATTAGCGGCCACATTCTCAATGAAC
	R330A	For – AGTGAAGCCAAAGTGCAGTTC<br>Rev – CACTTTGGCTTCACTTAATCCG
	M795A	For – ACACAGGCCGCAATGGACAAC<br>Rev – CATTGCGGCCTGTGTGACGG
	A1105W	For – CTGCAGTGGATCGCGCCCG<br>Rev – CGCGATCCACTGCAGCTGGTC
	A1105W/M795A	A1105W-For – CTGCAGTGGATCGCGCCCG<br>A1105W-Rev – CGCGATCCACTGCAGCTGGTC<br>M795A-For – ACACAGGCCGCAATGGACAAC<br>M795A-Rev – CATTGCGGCCTGTGTGACGG
TraI single and double site mutants in pHP2 for conjugation assay	Y190A	For – GCCAAAGGCAATCTGATTAGC**GGC**CACATTCTCAATGAACCCCGT<br>Rev – ACGGGGTTCATTGAGAATGTG**GCC**GCTAATCAGATTGCCTTTGGC
	R330A	For – CGTGTACGTGAACTGCACTTT**TGC**TTCACTTAATCCGGCAATCGC<br>Rev – GCGATTGCCGGATTAAGTGAA**GCA**AAAGTGCAGTTCACGTACACG
	M795A	For – CAGGGTGGCGTTGTCCATTGC**CGC**CTGTGTGACGGAGGCAAACAC<br>Rev – GTGTTTGCCTCCGTCACACAG**GCG**GCAATGGACAACGCCACCCTG
	A1105W	For – GAAAGACTGACCGGGCGCGAT**CCA**CTGCAGCTGGTCCGTGTCACC<br>Rev – GGTGACACGGACCAGCTGCAG**TGG**ATCGCGCCCGGTCAGTCTTTC
	A1105W/M795A	A1105W-For – GAAAGACTGACCGGGCGCGAT**CCA**CTGCAGCTGGTCCGTGTCACC<br>A1105W-Rev – GGTGACACGGACCAGCTGCAG**TGG**ATCGCGCCCGGTCAGTCTTTC<br>M795A-For – CAGGGTGGCGTTGTCCATTGC**CGC**CTGTGTGACGGAGGCAAACAC<br>M795A-Rev – GTGTTTGCCTCCGTCACACAG**GCG**GCAATGGACAACGCCACCCTG
